# Translational Control in Cardiac Pathophysiology and Therapeutic Development: When mRNA Meets the Heart

**DOI:** 10.3390/ijms26167863

**Published:** 2025-08-14

**Authors:** Uday K. Baliga, Liuqing Yang, Aleksandr Ivanov, Jack L. Schwartz, Feng Jiang, Eng-Soon Khor, Debojyoti Das, Lindsey Wainwright, Peng Yao

**Affiliations:** 1 Aab Cardiovascular Research Institute, Department of Medicine, University of Rochester School of Medicine & Dentistry, Rochester, NY 14586, USA; uday_baliga@urmc.rochester.edu (U.K.B.); liuqing_yang@urmc.rochester.edu (L.Y.); aleksandr_ivanov@urmc.rochester.edu (A.I.); jack_schwartz@urmc.rochester.edu (J.L.S.); fjiang7@stanford.edu (F.J.); darrenkhor90@gmail.com (E.-S.K.); debojyoti_das@urmc.rochester.edu (D.D.); lindsey_wainwright@urmc.rochester.edu (L.W.); 2 Department of Biochemistry & Biophysics, University of Rochester School of Medicine & Dentistry, Rochester, NY 14586, USA; 3 The Center for RNA Biology, University of Rochester School of Medicine & Dentistry, Rochester, NY 14586, USA; 4 The Center for Biomedical Informatics, University of Rochester School of Medicine & Dentistry, Rochester, NY 14586, USA

**Keywords:** cardiac regeneration, cardiomyocyte, congenital heart disease, fibroblast, fibrosis, heart failure, hypertrophy, mRNA translation, RNA-binding protein, RNA therapeutics

## Abstract

Cardiac physiology and pathology have been extensively explored at the transcriptional level. Still, they are less understood at the translational level, including three major knowledge gaps: pathophysiological impact, molecular mechanisms, and therapeutic implications of translational control in cardiac biology and heart disease. This review aims to provide a summary of the most recent key findings in this emerging field of translational control in heart health and disease, covering the physiological functions, disease pathogenesis, biochemical mechanisms, and development of potential RNA-based, translation-manipulating drugs. Translation of mRNA to protein is the final step in the central dogma for protein synthesis. Translation machinery includes a family of essential “housekeeping” factors and enzymes required for mRNA translation. These translation factors ensure the accurate processing of mRNA to protein according to the genetic code and maintain the optimal quality and quantity of cellular proteins for normal cardiac function. Translation factors also regulate the efficiency, speed, and fidelity of protein production and play a role in cardiac pathological remodeling under stress conditions. This review first introduces the techniques and methods used to study the translational regulation of gene expression in the cardiac system. We then summarize discoveries of a variety of pathophysiological functions and molecular mechanisms of translational control in cardiac health and disease, focusing on two primary symptoms, cardiac hypertrophy and fibrosis. In these sessions, we discuss the translational regulation directed by specific regulatory factors in cardiac physiology and how their genetic mutations, expression dysregulation, or functional alterations contribute to the etiology of heart disease. Notably, translational control exhibits extensive crosstalk with other processes, including transcriptional regulation, mitochondrial metabolism, and sarcomere homeostasis. Furthermore, recent findings have revealed the role of translational regulation in cardiomyocyte proliferation and heart regeneration, providing new approaches for creating regenerative medicine. Because transcript-specific translational regulation of both pathological and protective proteins occurs in heart disease, target-selective translation inhibitors and enhancers can be developed. These inhibitors and enhancers offer valuable insights into novel therapeutic targets and the development of RNA-based drugs for heart disease treatment.

## 1. Introduction

The heart is the organ responsible for pumping blood to supply oxygen and nutrients to other organs throughout the body. Cardiomyocytes (CMs) and cardiac fibroblasts (CFs) are the two major cell types in the heart. CMs handle the contractile function of the heart, while CFs stabilize the cardiac structure and repair potential cardiac damage. Cardiovascular disease is the leading cause of morbidity and mortality worldwide. Heart failure (HF), a major manifestation of cardiovascular disease, often results from cardiac ischemic events such as myocardial infarction (MI). Approximately 64 million people worldwide are affected by HF, with nearly half of these patients dying within five years of diagnosis [1]. An MI triggers proliferation and activation of cardiac fibroblasts; when left uncontrolled, this can lead to excessive cardiac fibrosis, reducing heart function and leading to HF. HF is clinically categorized based on ejection fraction (EF), which measures the percentage of blood pumped out from the heart with each heartbeat, into heart failure with reduced ejection fraction (HFrEF) and heart failure with preserved ejection fraction (HFpEF) [2]. HFrEF occurs when the left ventricle cannot contract properly and ejects less oxygen-rich blood after MI. Conversely, HFpEF maintains an EF above 50% but has impaired diastolic function and a reduced ability to fill the left ventricle with oxygenated blood, often developing after long-term comorbidities such as hypertension, obesity, and diabetes. Chronic HF remains the leading cause of hospitalization among patients over 65 years of age and poses significant clinical challenges and economic burdens both in the U.S. and globally.

**Figure 1:**
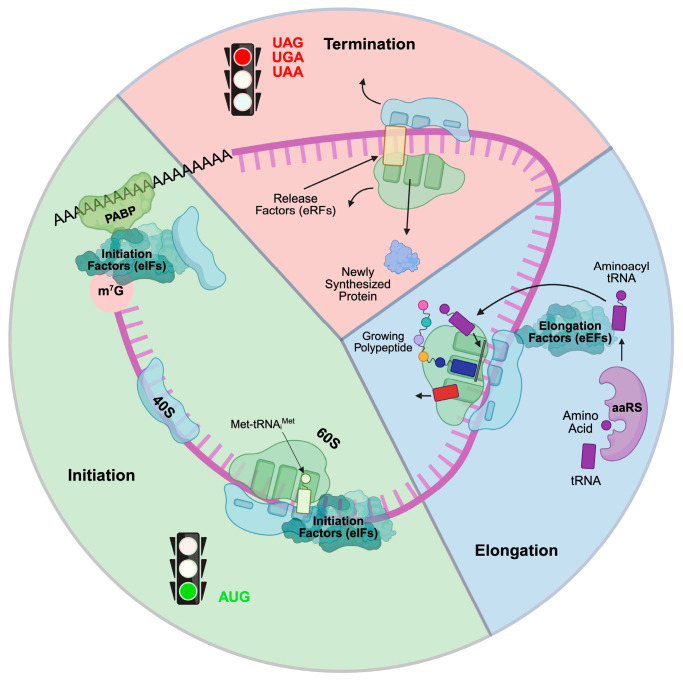
Overview of mammalian mRNA translation and essential components of the translation machinery.

The central dogma of molecular biology states that DNA is transcribed into mRNA (transcription), and mRNA is then translated into proteins (translation). Although transcriptional regulation has been studied extensively, relatively few investigations have focused on understanding the role of translational control in cardiac health and disease. Translation is crucial in producing functional proteins from mRNA transcripts in all biological processes (Figure 1), including cardiac cell proliferation, differentiation, hypertrophy, and fibrosis. While increased protein synthesis of pro-hypertrophic and pro-fibrotic genes has been observed during cardiac remodeling, these changes cannot be fully explained by transcriptional regulation alone. Transcription factors and microRNAs (miRNAs) are sequence-specific regulators of transcription and post-transcriptional processes (mRNA stability and translatability), respectively. The roles and mechanisms of transcription factors and miRNAs in cardiac biology have been extensively studied over the past twenty years. However, little is known about the molecular mechanisms of miRNA-independent translational control and the therapeutic potential of targeting the general translation process or transcript-specific translational regulation in the treatment of heart disease. Thus, understanding the abnormal translation control mechanisms that promote pro-hypertrophic and pro-fibrotic mRNA translation in cardiac cells is essential.

In eukaryotic cells, two sets of translation machinery exist: one in the cytoplasm and another within the mitochondria. Both systems comprise four major components: aminoacyl-tRNA synthetases (ARSs), translation factors, ribosomes, and translation-regulatory RNA-binding proteins (RBPs) (Figure 1). ARSs are a family of evolutionarily conserved housekeeping enzymes ubiquitously expressed across all three major domains of life. ARSs catalyze the ligation of amino acids to the 3′-terminus of cognate tRNAs bearing the correct anticodon triplet to ensure accurate decoding of mRNA into protein according to the genetic code [3,4]. Mammals have 20 cytoplasmic ARSs and 17 nuclear-encoded mitochondrial ARSs, with 3 ARSs shared between both cellular compartments. Typically, ARSs generally contain catalytic aminoacylation and tRNA anticodon recognition functions in separate domains. Several ARSs, including LARS1, IARS1, VARS1, AARS1, TARS1, FARS1, and EPRS1, contain dedicated editing domains for hydrolyzing mis-aminoacylated products to maintain translation fidelity [5,6]. Translation factors such as initiation, elongation, and termination factors are required for uploading, translocating, and disassembling ribosomal subunits, respectively. Cytoplasmic ribosomes comprise a 40S small subunit and a 60S large subunit, while mitochondrial ribosomes comprise a 28S small subunit and a 39S large subunit. All ribosomal subunits consist of numerous ribosomal proteins associated with ribosomal RNAs and are central for protein biosynthesis. RBPs and miRNAs bind to sequence- or structure-specific cis-acting elements and regulate the translation efficiency of target mRNAs. In pathological conditions, phenotypic changes are driven by the alteration of pathogenic proteins produced by the translation machinery. Notably, due to translational control, protein expression often does not correlate with mRNA abundance [7,8].

While prior reviews have extensively discussed RBPs and mRNA metabolism at the post-transcriptional level [9,10], this review focuses on understanding translational control in cardiac biology and heart disease. We begin by introducing various methodologies for studying translational control, including biochemical methods, deep sequencing approaches, and imaging-based techniques. Next, we discuss translational control in cardiac development and congenital heart disease, followed by an overview of its involvement in adult cardiac disease. Finally, we discuss therapies that target translational control mechanisms and outline future directions for investigations and therapies based on translational control in the heart.

## 2. Techniques and Methods to Investigate mRNA Translation in the Heart

### 2.1. Biochemical Methods for Studying Translational Control

#### 2.1.1. RNA-Binding Protein Immunoprecipitation (RIP) to Identify Bound Target RNAs

This method uses immunoprecipitation via specific antibodies to apply the pulldown of a specific target protein and its respective negative control [11,12]. Briefly, healthy growing cells or tissues (such as cardiac cells or the heart) undergo lysis with appropriate RNase and protease inhibitors, and cell or tissue lysates are prepared for immunoprecipitation. An immunoprecipitation-graded antibody for a target protein and a specific IgG isotype control are used in RIP experiments. Prior to immunoprecipitation, blocking and pre-clearing steps are performed to minimize nonspecific interactions. After pulldown, mRNAs and interacting proteins are separately extracted from the precipitated protein-RNA complexes. The isolated RNAs can be analyzed by quantitative PCR (qPCR) to assess specific RNA interactions, or by next-generation deep sequencing (RIP-seq) to determine the global RNA targets for the RNA-binding translation regulaotory proteins, such as YBX1 and CPEB4 (Table 1) [13,14]. The protein fraction is subjected to immunoblotting to confirm protein interaction or mass spectrometry for unbiased identification of the interactome of the target protein of interest.

#### 2.1.2. Crosslinking and Immunoprecipitation (CLIP) to Map RBP-Binding Sites on RNAs

This method examines the direct physical association between RNAs and their interacting proteins [15,16]. CLIP applies ultraviolet (UV) or formaldehyde crosslinking of live cells or tissues, which forms covalent bonds between RNAs and proteins in proximity [17,18]. Cell or tissue lysates are then prepared and subjected to partial fragmentation by a selected ribonuclease (e.g., RNase A or T1), followed by immunoprecipitation using a validated antibody for the target protein of interest and a corresponding IgG isotype control antibody. RNA fragments from the precipitated protein-RNA complexes are ligated with DNA adaptors on 3′ and 5′ ends (for individual-nucleotide resolution CLIP (iCLIP), DNA adaptors can be ligated on 5′ ends after reverse transcription) and purified by SDS-PAGE with size selection. The complexes are subjected to proteinase K digestion, and the recovered RNA fragments undergo reverse transcription to cDNA, DNA adaptor ligation on 5′ ends, and amplification via appropriate cycles of PCR reaction. Following the library construction, next-generation deep sequencing (CLIP-seq) is carried out to map transcriptome-wide protein binding sites on RNAs of a specific RNA-binding protein such as PRRC2B (Table 1) [15,19]. If a suitable immunoprecipitation-competent antibody for the target protein is unavailable, recombinant tagged RNA-binding proteins can be overexpressed, or transgenic tagging of endogenous RNA-binding proteins can be used for CLIP in a specific cell type.

#### 2.1.3. In Vitro Pulldown of Interacting Proteins of Biotinylated RNA

This technique uses a cell-free in vitro system to study RBPs and RNA interactions. Briefly, RNA is in vitro transcribed with internal biotin (biotinylated CTP) or 5′ biotin modification and incubated with cardiac cell or tissue lysates to capture interactions with specific RNA-binding proteins. The respective control oligo (scrambled or antisense oligo) and only beads are used as negative controls, and an immunoblotting experiment is conducted to detect any possible interactions [20]. Alternatively, mass spectrometry analysis can be undertaken to identify novel RNA-interacting proteins as an unbiased approach, followed by immunoblot confirmation (Table 1). This method has been used to determine the composition of the RNA-binding protein complex interacting with *VEGFA* mRNA 3′-UTR for regulating translation under hypoxic conditions [21,22].

#### 2.1.4. Proximity Ligation Assay Associated with Immunoblot or Mass Spectrometry

This technique applies selective biotinylating of proteins adjacent to a target protein of interest and can be used to study protein-protein interactions (PPI) [23,24] and protein-RNA interactions (RPI) [25] (Table 1). In brief, a biotin ligase is fused to a target protein of interest and expressed in living cells. Upon the addition of exogenous biotin to the culture media, the biotin ligase biotinylates proteins that potentially interact with the target protein. The biotinylated proteins are isolated using streptavidin beads and then coupled with mass spectrometry or immunoblot analysis to examine protein-protein interactions and create an interactome atlas of the target protein of interest. The mass spectrometry-based, label-free quantitative proteomics data (e.g., spectral count or intensity) can be scored using one of the SAINT (Significance Analysis of INTeractome) software packages to identify high-confidence protein-protein interactions (SAINT score > 0.95) [26].

#### 2.1.5. Puromycin Incorporation ASSAY to Assess Global Translation Efficiency

This is the most used technique for studying the protein synthesis status of cultured primary cells in vitro and animal tissues in vivo [27]. Cells are subjected to puromycin treatment for 15–20 min at 37 °C, followed by harvesting with a cell lysis buffer containing an appropriate protease inhibitor for protein isolation. The cellular protein lysates undergo immunoblotting analysis. Untreated cells are used as a negative control. In vivo puromycin incorporation enables the imaging of nascent proteins and the evaluation of translation regulation spatially and temporally in whole organisms [28]. An alkyne analog of puromycin, O-propargyl-puromycin (OP-puro), can form covalent conjugates with nascent polypeptide chains and label and visualize nascent proteins in the target organ of interest. This method is broadly applicable to imaging protein synthesis under both physiological and pathological conditions, both in vitro and in vivo (Table 1) [29,30].

**Table 1:** Overview of techniques and methods to investigate mRNA translation in the heart.

Technique and Method for Research in Translational Control	Purposes and Applications Related to Research in Cardiac Biology and Disease	Exemplary Reference
RBP immunoprecipitation (RIP)-seq	Identify target mRNAs of RBPs in cardiac cells	[13,14]
Crosslinking immunoprecipitation (CLIP)-seq	Map RNA binding sites recognized by RBP in cardiac cells	[15,19]
Biotinylated RNA pulldown	Identify RBPs interacting with RNA in vitro and in vivo	[21,22]
Proximity ligation assay	Identify protein-protein and protein-RNA interaction in cells	[24,25]
Puromycin incorporation assay	Measure nascent global protein synthesis in vitro and in vivo	[29,30]
Polysome profiling-seq	Determine mRNA translation efficiency in cardiac cells	[31,32,33]
Translating ribosome affinity purification-seq	Measure mRNA translation efficiency in specific cell types in vivo	[34,35,36]
Ribosome profiling (Ribo-seq)	Map ORFs and stalling sites in cardiac cells and whole hearts	[37,38,39]

### 2.2. Deep Sequencing-Based Translatome Profiling in Cells and Animals

#### 2.2.1. Polysome Profiling-Sequencing (Polysome-Seq)

Total cell or tissue lysates are subjected to a sucrose gradient solution and ultracentrifugation to separate different translation fractions, including free mRNP (mRNA-ribonucleoprotein complex), 40S ribosomal small subunits, 60S ribosomal large subunits, 80S monosomes, and polysomes (disomes, trisomes, and multiple ribosomes). Actively translated mRNAs bound by polysomes can be determined by next-generation deep sequencing to evaluate the translation efficiency of individual mRNAs as normalized by total RNA-seq signal [31]. Additionally, multiple pools can be collected, including non-polysome, monosome, and polysome pools, and their associated RNAs can be sequenced to calculate the distribution of specific mRNAs among different pools or individual fractions as translation efficiency, depending on the cost efficiency and demand for resolution (Table 1). This method has been employed to determine the translation efficiency at the transcriptome-wide level during cardiac fibroblast-to-myofibroblast activation and human embryonic stem cell (ESC)-to-cardiomyocyte differentiation [31,32,33].

#### 2.2.2. Translating Ribosome Affinity Purification Sequencing (TRAP-Seq)

Genetic engineering of endogenous ribosomal protein-coding genes in the mouse genome introduces a peptide or protein tag to the gene for subsequent affinity pulldown of ribosomes in vivo. Currently, three conditional inducible knock-in mouse models are available, including RPL22-3xHA, RPL10-EGFP, and mRPL62-FLAG [34,36,40]. In the former two cases, cytoplasmic ribosomes can be affinity-purified using HA or EGFP antibodies from tissue lysates to capture ribosomes from a specific cell type by immunoprecipitation of the large ribosomal subunit proteins RPL22 or RPL10 based on the use of the Cre recombinase transgenic mouse model. The latter targets the mitochondrial large ribosomal subunit protein mRPL62 for the purification of ribosomes in mitochondria. RNA-seq, following translating ribosome affinity purification (TRAP-seq), quantitatively measures translation efficiency in a specific cell type across various organs in vivo (Table 1). These two techniques were exploited to identify translationally regulated mRNAs in cardiomyocytes from both myocardial infarction and pressure overload-induced heart failure mouse models [34,35,36].

#### 2.2.3. Translational Landscape in Human and Mouse Heart Failure Determined by Ribosome Profiling (Ribo-Seq)

Ribosome profiling, also known as Ribo-seq, is a technique that measures the activity of ribosomes in translating mRNA into protein in a cell at a specific time. Ribo-seq utilizes deep sequencing to analyze ribosome-protected mRNA fragments after digesting the unprotected RNA surrounding the ribosome footprints with ribonucleases, such as micrococcal nuclease or RNase I. The sequenced ribosome-protected fragments can be used to determine the positions of translating ribosomes, define open reading frames (ORFs), measure the protein synthesis rate, map translation initation sites, examine translational elongation dynamics, and identify ribosome stalling sites in human and mouse hearts and culture cardiac cells (Table 1) [37,38,39]. Ribosome profiling provides a snapshot of ribosome density on individual mRNAs and translation activity. 

Our understanding of the translational control of gene expression in human diseases has always been sparse and less systematic due to the diversity of global and specific regulatory mechanisms. A recent study by van Heesch, S., et al. has attempted to solve this problem by generating a comprehensive landscape of translational regulation related to heart failure [37] using an integrative approach that combined RNA-seq, ribosome profiling (Ribo-seq), and mass spectrometry. Results were generated from 80 human hearts, including 65 failing and 15 non-failing hearts. This investigation provided a detailed assessment of translational control in the human heart, including regulation of translation by diverse cis- or trans-elements. By comparing RNA-seq and Ribo-seq results, the authors revealed translational downregulation of 327 genes and upregulation of 474 genes, including many cardiac disease marker genes, such as the extracellular matrix components genes associated with cardiac fibrosis. They also revealed specific cis-acting elements on mRNA correlated with translational regulation. For example, transcripts with a 5′ terminal oligopyrimidine (TOP) motif are significantly translationally upregulated, which agrees with previous studies about mTOR activation during heart disease. Apart from the 5′ TOP motif, upstream open reading frames (uORFs), another cis-acting element known to affect translation, have also been detected. For most proteins, the study found no clear correlation between the translation efficiency of the main ORF (mORF) and that of uORFs. However, several proteins, such as eIF4G2, exhibit an anti-correlation between the translation of the mORF and uORF, indicating a selective negative regulation of specific mRNAs via uORF in diseased human hearts at the endpoint. Alternatively, the correlation analysis using whole hearts at the endpoint of heart failure does not reveal the causal relationship between the translations of uORF and mORF due to the lack of temporal and spatial resolution, particularly regarding the early versus late stages of disease status and averaging effects from multiple cell types and anatomic locations.

They also detected a correlation between naturally occurring genetic variations and translation. They monitored the influence of single-nucleotide variants, insertions, and deletions on mRNA abundance, ribosome occupancy, and translation efficiency. Although most variations do not correlate with ribosome occupancy or translation efficiency, genetic association with translation is detected for around 100 genes. Notably, nonsense genetic mutations do not efficiently induce nonsense-mediated mRNA decay (NMD), as many protein-truncated variants (PTVs) are detected at the mRNA level. Ribosome occupancy of these PTVs shows little difference upstream or downstream of the premature stop codon, indicating inefficient termination or efficient re-initiation. The presence of these PTVs provides a possible cause of cardiac disease. Titin-truncating variants (TTNtvs) are detected in 13 dilated cardiomyopathy patients with different constitutive exons. No evidence of efficient NMD has been found, and significant ribosome occupancy downstream of the premature stop codon is detected in some cases, indicating that different TTNtvs may be translated differently and affect cardiac function in various ways. This observation partially explains the incomplete penetrance of heart disease occurrence in human patients with the same genetic mutation of TTNtvs. One possibility is additional expression and activity changes in eukaryotic release factors that may cause ribosome readthrough of premature termination codons but not native stop codons.

To complement Ribo-seq in whole human hearts, cell-type-specific translatomic analysis has been conducted. For instance, although post-transcriptional regulators such as IL-11 have been identified as related to cardiac fibrosis [8], the post-transcriptional mechanisms underlying myofibroblast transformation remained unexplored until 2019, when Chothani, S., et al. monitored the global changes in the transcriptome and translatome during human cardiac fibroblast activation and transformation to myofibroblasts using RNA-seq and ribosome profiling [41]. TGFβ was used to stimulate fibrosis in primary cardiac fibroblasts isolated from atrial biopsies of patients. Then, they performed RNA-seq and Ribo-seq at baseline and in a time-course ranging from 45 min to 24 h after TGFβ stimulation to capture a dynamic picture of the transcriptional and translational changes underlying the transition of quiescent fibroblasts into myofibroblasts.

They identified dynamically transcribed genes from RNA-seq and determined dynamically translated mRNAs from the integrated analysis of both RNA-seq and Ribo-seq results. A total of 1691 dynamically translated mRNAs were captured during the fibrotic response, of which translational changes alone were enough to cause changes in protein abundance. Sixty-seven dynamically translated mRNAs showed instant translational changes 45 min after TGFβ stimulation. The most enriched Gene Ontology term among those dynamically translated mRNAs was “transcription regulator activity,” suggesting that instant translational changes may modulate subsequent transcriptional changes. The impact of translation then gradually decreases at later time points while the effect of transcription gradually increases, indicating a possible shift from translational regulation to transcriptional regulation. However, although plenty of dynamically transcribed genes were detected, approximately 29% of the transcriptional changes are buffered by translational regulation, implying that translation efficiency may be upregulated on genes with limited transcripts or vice versa.

Chothani S. et al. suggest that over one-third of all gene expression changes detected in myofibroblast transformation involve translational regulation, which may be carried out through RNA-binding proteins (RBPs). During myofibroblast transformation, fifty-three differentially expressed RBPs were detected. The targets of these RBPs were predominantly enriched in dynamically translated genes, but not in dynamically transcribed genes, suggesting that these RBPs may shape the fibrotic response. However, loss-of-function and gain-of-function validation studies for individual RBPs are required to form the causal relationship between these RBPs and the fibrotic response.

### 2.3. Imaging-Based Techniques for Evaluating Translation Efficiency and Localized Translation in Cardiomyocytes

Puromycin, an unnatural analog of tyrosyl-tRNA, can be incorporated into the aminoacyl (A) site of translating ribosomes, leading to truncated protein production terminated at this residue. Treatment of cultured cardiac cells with puromycin can be used to evaluate the global protein synthesis rate using immunoblot. Moreover, injecting puromycin into animals can facilitate the direct imaging of translation events in vivo. This method can provide information on the location of translation in organs and allow for the quantification of translation efficiency by comparing control and genetically modified or disease-triggering animal models. One example is the visualization of translation machinery colocalized with the sarcomere protein network in mouse cardiomyocytes in vivo [42]. Macromolecular protein complexes, such as the sarcomere—the basic contractile macromolecular complex of cardiomyocytes—are maintained with proper localization and fixed subunit stoichiometry. Single-cell analysis of cardiomyocytes using mRNA and protein synthesis imaging demonstrates three different but related mechanisms for retaining the sarcomere [42]: (i) Mature mRNAs encoding sarcomere component proteins are localized to the sarcomere where their protein products are assembled. (ii) Translation machinery, such as ribosomes, is located at the sarcomere with localized translation of sarcomere protein-coding mRNAs. (iii) A specific, localized E3 ubiquitin ligase enables the rapid and efficient degradation of excess, unincorporated sarcomere component proteins. These three mechanisms are distinct and required. Cooperation of the mechanisms is essential to ensure appropriate spatial localization of sarcomere proteins and to buffer the variability in mRNA expression levels of these proteins. Cardiomyocytes maintain their sarcomeres through localized translation at high rates and continuous proteasomal degradation, which removes excess proteins and maintains the homeostatic stoichiometric ratio of different component proteins within the sarcomere protein network. Therefore, tightly regulated localization of mRNA transcripts, translation, and protein degradation controls the organization of sarcomere assembly and maintains the spatiotemporal features.

During cardiac hypertrophy, stress-induced signal transduction enhances mRNA translation in cardiomyocytes, facilitating the addition of new contractile sarcomere units, which enlarges cell size and enhances contractile function. In this process, microtubules are required for cardiomyocyte growth via spatiotemporal control of the translation machinery [43]. In particular, Scarborough et al. show that microtubule motor protein Kinesin-1 localizes ribosomes and mRNAs along microtubule tracks to different regions within the cell. Microtubules normally deliver mRNAs and translation machinery to specific sites, promoting local translation and the assembly of cardiomyocyte contractile units. This microtubule network is disrupted by a hypertrophic stress stimulus (phenylephrine treatment), leading to the collapse of ribosomes and mRNAs around the nucleus, resulting in mislocalized protein synthesis and rapid degradation of newly synthesized proteins. Consequently, cardiomyocytes fail to grow despite increased translation rates, suggesting that properly localized translation, not just the translation rate, is a key determinant of cardiac hypertrophy.

The synthesis of sarcolemma and sarcoplasmic reticulum membrane-associated proteins in cardiomyocytes was assumed to follow the general secretory pathway, with localized mRNA translation occurring in perinuclear areas, followed by protein trafficking and delivery to functional sites. However, limited experimental evidence was provided. Using a single-molecule level visualization and a proximity-ligated in situ hybridization approach, researchers visualized ribosome-associated mRNAs for ion channel-related proteins, such as SERCA2A and SCN5A, providing detailed information on the localized translation sites within the cell [44]. The translation machinery for membrane-associated protein synthesis occurs throughout the cardiomyocyte, enabling the distributed synthesis of specific transmembrane proteins within specific subcellular locations. In these niches, localized ribosomes synthesize proteins from local mRNA pools, which are trafficked from the nucleus by association with the microtubules and cytoskeleton network. As an evolutionarily conserved mechanism from mouse to human, membrane protein mRNAs are widely distributed across the cardiomyocyte in normal and failing human heart tissues. These findings confirm that local protein synthesis in cardiomyocytes plays a crucial role in regulating cardiac structure and function. At the pathophysiological level, arrhythmias and sudden death can occur with a mild imbalance between inward sodium and outward potassium currents. A new paradigm offers insights into the mechanisms that maintain this critical balance. Electrophysiological and single-molecule fluorescence imaging analysis reveal that two mRNAs encoding SCN5A (I_Na_) and hERG (I_Kr_) channels are associated and coordinated in defined, discrete complexes, namely, “micro-translatomes”, during protein translation [45]. Approximately half of the hERG-translating complexes contain *SCN5A* mRNA transcripts. Moreover, both mRNA transcripts are regulated at co-translational levels, and consequently, this regulation alters the expression of both functional ion channels localized at the cytoplasmic membrane.

## 3. Translational Control in Cardiac Development and Congenital Heart Disease

### 3.1. Human Genetic Mutations in Translation Machinery and Congenital Heart Disease

#### 3.1.1. Diamond Blackfan Anemia and Other Heart Disease-Causing Mutations in Cytoplasmic Translation Factors

**Figure 2:**
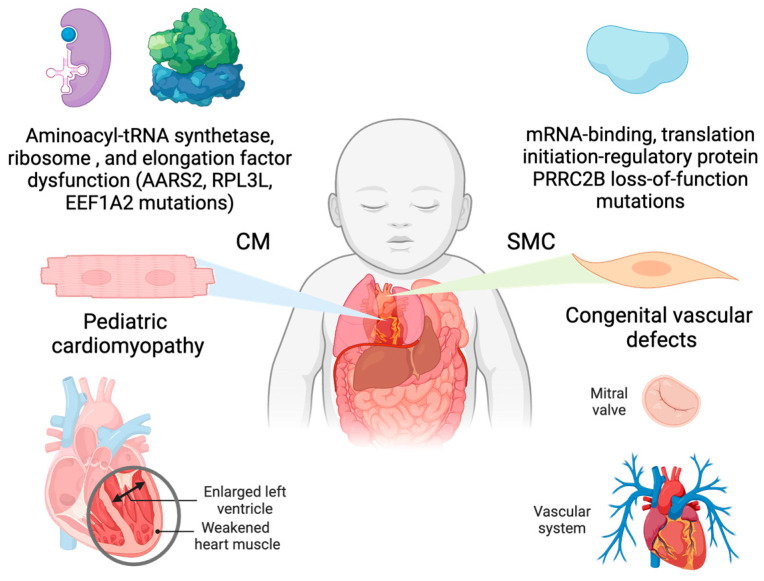
Translation machinery defects in early-onset human heart disease. Loss-of-function mutations in mitochondrial alanyl-tRNA synthetase (AARS2), large subunit ribosomal protein 3-like (RPL3L), and eukaryotic translation elongation factor 1A2 (EEF1A2) cause early-onset cardiomyopathy and heart failure. Premature termination codon or intronic splicing site mutation in an RNA-binding protein and translation-regulatory factor, proline-rich coiled coil protein 2B (PRRC2B), are associated with mitral valve and vascular diseases in congenital heart disease probands.

Human genetic mutations in cytoplasmic large ribosomal subunit protein 5 (RPL5), among many other cytoplasmic ribosomal protein-coding genes, lead to Diamond Blackfan Anemia with congenital cardiac developmental defects [46,47,48], suggesting that loss-of-function mutations in ubiquitously expressed housekeeping translation factors can result in cell-type- and organ-specific disorders. This observation has been well recapitulated in mouse and zebrafish genetic models with ribosomal protein mutations [49]. Most of these ribosomal proteins are expressed ubiquitously across organs and cell types, such as RPL5 and RPL3 (large ribosomal subunit protein 3). Intriguingly, multiple ribosomal protein paralogs are expressed in a tissue-specific manner. It is under debate how these proteins influence translation in specific organs and affect the development and function, such as the heart. Large ribosomal subunit protein 3-like (RPL3L), a paralog of RPL3, is specifically expressed in cardiomyocytes and skeletal muscle cells of the heart and skeletal muscle, respectively, and modulates the dynamics of the translation elongation process. Genetic mutations of RPL3L in humans are associated with pediatric cardiomyopathy and age-related atrial fibrillation [50] (Figure 2). To recapitulate the genetic defects in human patients, a homozygous *Rpl3l* knockout mouse model has been established in multiple labs. Shiraishi’s group reported that a deficiency of RPL3L-bearing ribosomes in *Rpl3l* global knockout mice (CRISPR-Cas9-mediated deletion of the exon 2) resulted in reduced cardiac contractility [51]. Transcriptome-wide ribosome profiling assay showed that ribosome occupancy at mRNA genetic codons was changed in the *Rpl3l*-null heart, with changes negatively correlated to those observed in myoblast cells with RPL3L overexpression. Compared with RPL3-bearing canonical ribosomes, RPL3L-bearing ribosomes were less prone to ribosome collisions on the mRNA. The reduction of RPL3L-containing ribosomes reprograms the translation elongation dynamics for the global transcriptome. Intriguingly, this translation-altering effect is most significant for mRNA transcripts encoding proteins related to cardiac muscle contraction and dilated cardiomyopathy, where the quantity of these proteins is decreased due to repressed translation. Thus, RPL3L-bearing ribosomes are essential to maintain the translation elongation dynamics required for normal cardiac function. This finding provides insights into the mechanisms of tissue-specific ribosomal protein-mediated translational regulation with physiological and pathological relevance in human patients.

In contrast, Milenkovic’s group recently demonstrated a dynamic interplay between RPL3- and RPL3L-bearing ribosomes that regulates mitochondrial activity and ATP production in the mammalian heart as a translation-independent noncanonical mechanism. Different cell types possess distinct types of ribosomes, each with specialized ribosomal proteins, such as RPL3L. This phenomenon is defined as ribosome heterogeneity. However, whether this ribosome heterogeneity results in functionally diverse “specialized ribosomes” in canonical mRNA translation or noncanonical functions remains controversial. Milenkovic’s group used a similar but different *Rpl3l* knockout mouse strain (CRISPR-Cas9-mediated deletion of a 13-bp DNA fragment in the exon 5) to uncover a rescue mechanism in which compensatory induction of RPL3 is triggered upon RPL3L inactivation, accumulating RPL3-bearing ribosomes instead of RPL3L-bearing ribosomes that are uniquely present in cardiomyocytes [52]. In contrast to Shiraishi’s group’s findings, using ribosome profiling and a novel approach of ribosome pulldown coupled with nanopore RNA-seq (Nano-TRAP), RPL3L is found to modulate neither translational efficiency nor ribosome affinity towards a specific subset of mRNA transcripts. Interestingly, the knockout of *Rpl3l* leads to enhanced ribosome–mitochondria interactions and a significant increase in mitochondrial activity, resulting in increased ATP synthesis in CMs. This study suggests that tissue-specific ribosomal protein paralogues may not regulate the translation of specific mRNA transcripts. Alternatively, the presence or absence of RPL3L alters the expression of RPL3, which in turn changes the subcellular localization of cytoplasmic ribosomes and modifies mitochondrial activity. What factors cause the discrepancy between these two studies, which use similar approaches, remains unclear. One of the most reproducible findings suggest that unrelated genetic deletion distinct from *Rpl3l* loss-of-function, such as conditional knockout of a cytosolic gene, glutamyl-prolyl-tRNA synthetase (*Erps1*), or a nuclear-encoded mitochondrial gene family with sequence similarity 210 member A (*Fam210a*) in CMs, caused heart failure and exhibited simultaneous increase in RPL3 and decrease in RPL3L mRNA and protein expression levels [31,39]. Cryo-electron microscopy visualization of RPL3- and RPL3L-bearing ribosome structures, along with comparative bioinformatic re-analysis of independent Ribo-seq data from both groups, will provide critical insights into deciphering the functional and mechanistic divergence between the two specialized ribosomes in the CMs.

A recent report from Molkentin’s group showed that mouse cardiac ventricles express RPL3 during the neonatal stage [50]. RPL3 is then replaced by RPL3L in adulthood, but it is re-expressed under conditions of cardiac hypertrophy and remodeling. This follows a similar expression pattern to the fetal gene program, including key transcription factors and sarcomere proteins. Intriguingly, *Rpl3l*^−/−^ mice (CRISPR-Cas9 mediated deletion and frameshifting in the exon 5) showed no overt changes in cardiac structure or function at baseline or after transverse aortic constriction surgery-based, pressure overload-induced hypertrophy. Possibly, loss-of-function of RPL3L could be compensated by RPL3, as the expression of the latter was persistently upregulated in the adult heart [50]. Transcriptomic profiling analysis and polysome profiling assays reveal little difference between *Rpl3l* knockout and wild-type control hearts from adult mice. Moreover, in adult *Rpl3l* knockout cardiomyocytes, no changes were found in cellular localization of the ribosome, cardiac tissue ultrastructure, or mitochondrial function compared to wild-type control cells. Adeno-associated virus-9 (AAV9)-mediated overexpression of either RPL3 or RPL3L in the hearts of mice failed to induce pathogenesis. *Rpl3l* null mice had significantly smaller hearts during cardiac aging than wild-type controls at 18 months after birth. Unlike the other two groups, Molkentin’s lab demonstrates that *Rpl3l* knockout can be fully compensated by RPL3, although *Rpl3l* deletion leads to a slight but significant reduction in heart weight. However, no rigorous ribosome profiling was conducted to provide any insights into gene dysregulation at the translational level. More replicated studies are required to reproduce and confirm these findings, as the different genetic knockout strategies employed by the three labs may partially contribute to the contradictory conclusions and distinct phenotypes and mechanisms. In addition, ribosome profiling (Ribo-seq) mapping is necessary to provide detailed information about subtle changes in translation elongation dynamics that polysome sequencing (polysome-seq) cannot. To resolve this controversy, genetic knock-in of the specific human mutation in the mouse genome needs to be performed to recapitulate the heart disease phenotype observed in human patients. If no spontaneous phenotype is triggered, it suggests the possibility of human mutations as accompanying or modifier variants, which may not directly cause symptoms of heart disease.

Intriguingly, a preprint manuscript from Wu’s lab provided further mechanistic insights into how PRL3L mutant proteins lead to dilated cardiomyopathy 2D (CMD2D; OMIM #619371) due to a gain of toxic function in humans [53] (Figure 2). They identified new, rare yet highly pathogenic heterozygous hotspot mutations D308V/N and G27D in the *RPL3L* gene. These mutations were co-initiated with known loss-of-function mutations (frameshift, missense mutation, alternative splicing) or low-pathogenic missense mutations. Despite carrying autosomal recessive alleles, such patients do not exhibit severe heart failure. The authors observed a decrease in 28S rRNA but not 18S rRNA in the tissue of these patients. Moreover, neither *RPL3* mRNA nor protein levels were found to be upregulated as a compensatory response. To evaluate the impact of each mutation, the authors developed an AC16 human cardiomyocyte cell model with inducible RPL3 and different variants of RPL3L to assess the effect of each mutation. D308V/N and G27D variants of RPL3L showed a reduction in 28S rRNA and 60S ribosomal subunits, resulting in the loss of 80S monosomes and polysomes. Being heterozygous and recessive, the mutations confer a gain of toxic function rather than a loss of function. The D308V/N and G27D RPL3L proteins were detected solely in the nucleus. Interactome research revealed their interaction with proteins involved in 28S rRNA processing and 60S ribosomal subunit biogenesis. The nuclear-localized mutant RPL3L exhibited the highest binding affinity with the ribosomal chaperones and biogenesis factors GRWD1 and C7ORF50, as indicated by HA-tagged RPL3L overexpression followed by immunoprecipitation and mass spectrometry analysis. Thus, D308N and G27D proteins may sequester ribosome biogenesis factors, ultimately diminishing overall translation. Additionally, the authors found that the R161W and T189M mutants of RPL3L induced a compensatory effect on RPL3, mirroring the upregulation of *Rpl3* mRNA in the *Rpl3l* knockout mouse model. These two mutations enhance the stability of *RPL3* mRNA without affecting its transcription. However, the compensation from increased RPL3 was inadequate to rescue the failure caused by the gain-of-function mutation allele of *RPL3L*. Publicly available human genome sequencing databases reveal more than ten individuals with homozygous RPL3L knockouts, with no reported cases of heart disease. This supports the idea that the RPL3L mutations have a gain of toxic function and genetic knockout of *RPl3l* in mice may not necessarily cause heart diseases as observed in *RPL3L*-null humans. Noticeably, they used lentiviral overexpression of mutant RPL3L with shRPL3 knockdown in an immortalized human AC16 ventricular cardiomyocyte cell line fused with human fibroblast cells. This experimental system may not necessarily recapitulate the gene expression and protein localization in vivo in the human heart. Genetic knock-in mouse model for the human RPL3L mutations and human iPSC-CM after maturation need to be exploited to validate their findings in the AC16 cell line. Additionally, the mechanism underlying the stabilization of *RPL3* mRNA by the mutant RPL3L protein remains unclear and warrants further thorough biochemical characterization.

In a similar case, a gain-of-toxic-function human mutation, S637G, in RBM20 (RNA binding motif protein 20), leads to the mislocalization of this protein in the cytoplasm, the formation of RBM20-ribonucleoprotein granules, and thereby causes severe dilated cardiomyopathy by inhibiting mRNA translation, most likely. The severe heart failure symptoms and high mortality rate are faithfully recapitulated in a *Rbm20*^S639G^ knock-in mouse model, but not in the *Rbm20* knockout model with a mild heart disease phenotype [54]. These findings may be inspirational for a better understanding of RPL3L biology. In human patients, the toxic gain-of-function of PRL3L driven by missense mutations likely causes severe heart disease symptoms, which cannot be recapitulated by *Rpl3l* knockout in mice. This supports the necessity of generating global homozygous knock-in animal models to faithfully replicate multiple loss-of-function or gain-of-function missense mutations in human patients. Spontaneous cardiac disease phenotypes can be characterized in depth to compare with the patients’ corresponding symptoms. Notably, gene knockout models using different genetic manipulation strategies often exhibit distinct phenotypic outcomes caused by alternative splicing or translation reinitiation [55,56,57]. Most importantly, ribosome profiling needs to be carried out at different developmental time points to dissect the translation dynamics throughout embryonic, prenatal, postnatal, and aging stages in the *Rpl3l* knockout mouse model compared to the wild-type controls [37]. Alternatively, the knock-in of an epitope tag (such as HA or FLAG) at the C-termini of RPL3 and RPL3L protein-coding genes, followed by RiboTag immunoprecipitation and deep sequencing [36], will enable the identification of their selective mRNA targets during translation by these two specialized ribosomes. In the long term, cryogenic electron microscopy (cryo-EM), a transmission electron microscopy technique, can be utilized to determine the structural variation between RPL3L- and RPL3-bearing ribosomes and structure-function relationships at the atomic level [58].

Unbiased animal genome- and transcriptome-wide associated studies (GAWS and TWAS) and subsequent expression quantitative trait locus (eQTL), as well as translation efficiency quantitative trait locus (teQTL) provide a complementary approach to human GWAS, TWAS, eQTL, and subsequent characterization of the causative genetic variants of diseases. One such study identified a trans locus that causes ribosomopathy in hypertrophic hearts, modulating mRNA translation in a protein length-dependent manner through teQTL analysis in rats [59]. This study investigated the influence of trans-acting genetic variation in distant genetic loci on the mRNA translation efficiency. It defined their contribution to the development of complex disease phenotypes within a panel of rat inbred lines. One of the tissue-specific master regulatory loci, explicitly associated with hypertrophic hearts, drives transcriptome-wide protein length-dependent regulation of mRNA translation efficiency. This alters the stoichiometric translation rates of many sarcomere protein-coding mRNAs. Mechanistically, significant differences in global polysome profiles and dysregulation of the small nucleolar RNA *SNORA48* influence ribosome biogenesis and activity, leading to a translation machinery defect. Reproducible protein length-dependent shifts in translational efficiency were observed as an evolutionarily conserved trait of translation machinery mutants across multiple species from yeast to humans, including ribosomopathy-causing translation machinery component protein mutants. Mutations in different trans-acting factors can reduce or enhance a negative correlation between protein length and translation rates. This effect is potentially caused by imbalances in transcript-specific translation initiation and re-initiation rates.

Another ribosome-related translation component is eukaryotic elongation factor 1A (eEF1A). eEF1A mediates aminoacyl-tRNA recruitment to the aminoacyl-tRNA site (A-site) of the eukaryotic cytoplasmic 80S ribosome. Therefore, eEF1A is crucial to the translation elongation process on mRNAs. Two different isoforms exist, eEF1A1 and eEF1A2, though antibodies to differentiate these isoforms have only recently become available. eEF1A1 is ubiquitously expressed through embryonic and neonatal mice and is then reduced and replaced by eEF1A2 in the heart, brain, spinal cord neurons, and skeletal muscle. Mice with a 15.8 kilobase deletion in the *Eef1a2* gene have a “wasted” phenotype, experiencing muscle wasting, neurodegeneration, and death at postnatal day 28 [60]. Humans with a Pro^333^-to-Leu mutation in the *EEF1A2* gene have dilated cardiomyopathy, developmental delay, epilepsy, and early death [61] (Figure 2). *Xmlc2*-Cre^+^ driven cardiomyocyte-specific conditional knockout of *Eef1a2* and knock-in of Pro^333^-to-Leu mutation in mice resulted in left ventricular chamber dilation and systolic dysfunction, followed by full penetrance of death at around 8–17 weeks [62], suggesting an essential role of eEF1A2 in heart development and functional maintenance. Like RPL3L and RPL3, it is crucial to dissect the functional redundancy and uniqueness between eEF1A1 and eEF1A2, as well as their role in the heart at fetal, neonatal, and postnatal stages, as a future direction.

#### 3.1.2. Human Mutations in Mitochondrial Translation Machinery Lead to Genetic Cardiomyopathy

Numerous human mutations in mitochondrial translation machinery have been reported to cause spontaneous early onset dilated or hypertrophic cardiomyopathy. These cardiomyopathy-related mitochondrial translation machinery mutations include mitochondrial aminoacyl-tRNA synthetases and tRNAs, translation factors, mitochondrial ribosomal proteins, and mitochondrial RNA-binding proteins [63]. An illustration of this is a mutation in the mitochondrial alanyl-tRNA synthetase gene, AARS2, resulting in infant-onset cardiomyopathy [64,65,66] (Figure 2). This implies that AARS2-dependent translation is necessary for normal cardiac development. Another example of mitochondrial translation affecting cardiomyopathy was shown in experiments by Rudler et al. [67]. They generated a knockout mouse model of *Mtif3*, a mitochondrial translation initiation factor. A global knockout mouse model of *Mtif3*^fl/fl^ with a universal Cre transgene was embryonic lethal. In contrast, a heart and skeletal muscle-specific knockout mouse model with *Ckmm*-Cre resulted in dilated cardiomyopathy. Evidence of mutations in mitochondrial translation machinery component genes resulting in cardiomyopathy highlights the importance of normal mitochondrial translation in maintaining mitochondrial integrity and cardiac function.

#### 3.1.3. Loss-of-Function of PRRC2B-Mediated Translation Initiation Regulation Causes Congenital Cardiovascular Defect in Humans and Mice

**Figure 3:**
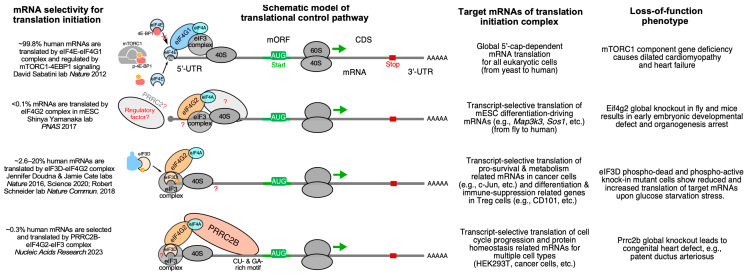
eIF4G2-PRRC2B complex-mediated translation initiation regulatory pathway for cell proliferation and cardiac developmental integrity. The pathway of eIF4G2-PRRC2B-directed translation initiation of a specific cohort of mRNAs encoding cell cycle progression and protein homeostasis-related proteins (**bottom row**) is compared to the cap-dependent, eIF4G1-eIF4E-driven canonical translation initiation (**top row**), cap-independent, eIF4G2-mediated (**second row**), and cap-dependent, eIF4E-independent, eIF3D-mediated (**third row**) noncanonical translation initiation pathways. Green arrow: ribosome movement during translation elongation. Question mark: unaddressed questions regarding the unknown regulatory factor, protein-protein and protein-RNA interactions for future studies.

Posttranscriptional control of gene expression, including RNA splicing, transport, modification, translation, and degradation, is primarily mediated by RBPs through their interactions with target mRNAs [68]. Recently, our group characterized the function of a novel RBP, Proline-rich coiled-coil 2B (PRRC2B) [15] (Figure 2). Transcriptome-wide CU- or GA-rich RNA regions were identified as PRRC2B binding sites near the translation initiation codon on a specific cohort of mRNAs by photoactivatable ribonucleoside-enhanced crosslinking and immunoprecipitation and sequencing (PAR-CLIP-seq) in human cells. These PRRC2B-bound mRNAs, including oncogenes, proteins involved in maintaining cellular homeostasis, and cell cycle regulators such as cyclin D2 (*CCND2*), showed decreased translation efficiency when PRRC2B was inducibly knocked down, leading to disrupted G1/S phase transition and impaired cell proliferation. Antisense oligonucleotides (ASOs) that block PRRC2B interaction sites within the *CCND2* mRNA 5′-UTR reduced its translation and hindered cell cycle progression and growth. Mechanistically, PRRC2B interacts with eukaryotic translation initiation factors 4G2 (eIF4G2) and eIF3 independently of RNA, forming a ribonucleoprotein complex with target mRNAs. The interaction between PRRC2B and eIF4G2 is critical for sufficient translation initiation of *CCND2* mRNA. Thus, PRRC2B is a vital translation regulatory factor for the efficient expression of a selective group of proteins (~0.3% of human genes) essential for cell cycle progression and proliferation (Figure 3). We confirmed that shRNA-mediated knockdown of PRRC2B inhibits translation of a specific set of endogenous mRNAs in human cells, including *CCND2* [15]. Interestingly, CCND2 protein has been reported to promote cardiomyocyte proliferation when overexpressed in pig hearts, suggesting its potential role in enhancing heart regeneration in large mammals [69], which implies that PRRC2B may be involved in cardiomyocyte proliferation at the translational level. In line with this idea, Teleman’s group reported the critical role of PRRC2 family proteins—specifically PRRC2A, PRRC2B, and PRRC2C—in the proliferation of human cancer cells [70]. Notably, our work and several earlier studies highlight the diversity of translation initiation mechanisms across different cell types and stress conditions, including mTORC1-regulated eIF4G1-eIF4E-mediated canonical translation initiation conserved from yeast to humans [71], eIF4G2-directed cap-independent translation initiation in mouse embryonic stem cells [72], as well as eIF4E-independent, cap-dependent, eIF3D-driven [73,74,75] or eIF4G2-eIF3D complex-mediated [27,76,77] translation initiation pathways. 

Our recent work highlighted a novel function of PRRC2B associated with congenital heart disease [78]. We identified two alternatively spliced isoforms of PRRC2B and confirmed their conservation in human and mouse hearts, as well as in HEK293T cell lines. Kimchi’s group recently reported the same alternative spliced isoforms in multiple additional human cancer cell lines, such as HeLa, HCT116, and A549, among others [79]. Interestingly, our in vivo exon-16-containing premature termination codon knock-in mouse model for full-length PRRC2B did not show any severe cardiac phenotype, suggesting a possible compensation either by the alternate spliced isoform (exclusion of exon 16, as termed ΔE16) or possibly by PRRC2A/PRRC2C paralogs. However, global knockout of both full-length and ΔE16 *Prrc2b* mRNA isoforms (genetic deletion of exon 4, which is shared by both isoforms) causes high penetrance of neonatal lethality in mice due to patent ductus arteriosus (PDA), a congenital cardiovascular developmental defect also observed in humans. Bulk and single-nucleus RNA-seq analyses from *Prrc2b* knockout hearts revealed a potential decrease in smooth muscle cell (SMC) populations or downregulation of SMC-specific genes. Moreover, polysome-seq, RNA-seq, and mass spectrometry analysis from CRISPR-Cas9-mediated PRRC2B knockout HEK293T cells suggest a significant reduction of genes involved in cell proliferation, heart and vascular development, indicating a possible regulation of PRRC2B in vascular smooth muscle cell proliferation and contraction. Two heterozygous loss-of-function mutations of the *PRRC2B* gene in patients with congenital heart disease are reported to manifest symptoms of pulmonary vein atresia and mitral regurgitation and stenosis, underscoring the connection between PRRC2B and cardiovascular development and disorders. Therefore, PRRC2B has been hypothesized to regulate the protein translation of specific proteins in cardiac cells, such as smooth muscle cells, to maintain normal heart morphogenesis. Our dual-omics analysis (translatome and proteome) of E18.5 embryonic hearts from global *Prrc2b* knockout and wild-type control mice, including polysome-seq and mass spectrometry, suggests that the loss of function of PRRC2B causes aberrant gene expression in cell proliferation, migration, and artery development, thereby leading to congenital heart defects. Our recent findings indicate that the knockdown of *PRRC2B* in primary human aorta-derived smooth muscle cells inhibited cell proliferation and migration [78] (Figure 3). Importantly, analysis of clinical data on PRRC2B mutations in 3740 probands with congenital heart disease indicated a significant association with atrial septal defect [78]. Another group reported the role of PRRC2B in cerebral vascular remodeling under acute hypoxic conditions through both N6-methyladenosine (m6A)-dependent and m6A-independent posttranscriptional regulation using an endothelial cell-specific conditional knockout mouse model [80]. Therefore, PRRC2B is an emerging RNA-binding protein that plays a pivotal role in the development of cardiovascular disease and could serve as a future therapeutic target. As a next step, deep phenotyping of the global *Prrc2b* knockout mice and a future smooth muscle cell-specific inducible *Prrc2b* knockout mouse model will be required to robustly validate the function of PRRC2B in vivo.

#### 3.1.4. eIF4E1C Regulates Cardiomyocyte Metabolism and Proliferation During Heart Regeneration in Zebrafish

In eukaryotes, the eIF4E family of translation initiation factors, such as canonical eIF4E1A, bind 5′ methylated guanosine caps of mRNAs as a limiting step for mRNA translation. Another family member, eIF4E1C, present in aquatic vertebrates but lost in terrestrial species, plays a vital role during cardiac development and heart regeneration in zebrafish [81]. eIF4E1C is broadly expressed across multiple cell types and organs in fish. Genetic deletion of the *eif4e1c* gene in zebrafish caused growth defects and reduced juvenile survival. The knockout zebrafish surviving to adulthood had a significantly decreased number of cardiomyocytes and compromised proliferation in response to cardiac injury compared to wild-type controls. Translatome-wide Ribo-seq analysis of *eif4e1c*-null hearts reveals changes in the translation efficiency of mRNAs encoding proteins that regulate cardiomyocyte cell proliferation. Disruption of eIF4E1C function in the mutant fish had the most pronounced impact on the heart at juvenile stages. This suggests a specialized requirement for fine-tuning the control of translation initiation via a unique eIF4E paralog in fish for heart regeneration and development. However, identifying a similar translation factor and regulatory mechanism in terrestrial species (e.g., mammals) is the key to generalizing this concept in evolution.

### 3.2. Translational Control in Mitochondrial Cardiomyopathy

Many genetic mutations have been reported in the genes encoding mitochondrial translation machinery that cause spontaneous cardiomyopathy with mitochondrial dysfunction, including mitochondrial aminoacyl-tRNA synthetases and tRNAs, mitochondrial translation initiation and elongation factors, mitochondrial ribosomal proteins, and other translation regulatory factors or RNA-binding proteins localized in the mitochondria [63]. Persistent activation of an evolutionarily conserved central cytosolic translational control pathway, namely the integrated stress response (ISR), is considered a common shared feature among multiple types of mitochondrial cardiomyopathy and other mitochondrial dysfunction-related diseases caused by mutations in these mitochondrial protein-coding genes [82,83].

**Figure 4:**
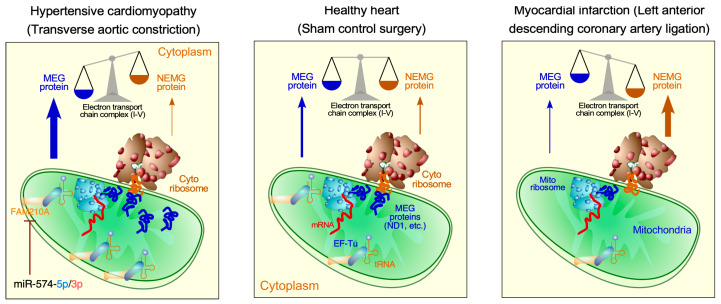
FAM210A-EF-Tu complex regulates mitochondrial translation elongation and cardiac mitochondrial homeostasis in hypertensive cardiomyopathy and ischemic heart failure. **Left panel**: miR-574-5p and miR-574-3p downregulate FAM210A expression and limit cardiac hyper-trophy in transverse aortic constriction-induced hypertensive cardiomyopathy. **Middle panel**: optimized level of FAM210A recruits EF-Tu, a mitochondrial translation elongation factor, to maintain the balance between the cytosolic and mitochondrial translation, thereby preserving protein homeostasis. **Right panel**: FAM210A expression is reduced in left anterior descending coronary artery ligation-induced myocardial infarction, leading to pronounced mitochondrial dysfunction and contributing to heart failure.

Translational regulation within mitochondria remains underexplored, and further research is necessary to identify novel therapeutic targets in the translational process for treating mitochondrial diseases. Prior genome-wide association studies in humans have revealed that non-coding region mutations in the *FAM210A* (family with sequence similarity 210 member A) gene are associated with skeletal muscle disorders and bone fractures [84,85]. We recently showed that the *Fam210a* cardiomyocyte-specific genetic knockout mouse model exhibited progressive mitochondrial cardiomyopathy and heart failure [39]. Multi-omics analyses, including RNA-seq, Ribo-seq, proteomic mass spectrometry, and metabolomics, revealed a reduction in mitochondrial-encoded mRNA translation elongation and consequent activation of ISR in *Fam210a* knockout hearts. Chronic and persistent ISR activation inhibits cap-dependent translation initiation in the cytoplasm, leading to translational reprogramming and disrupted protein homeostasis. Interestingly, FAM210A protein expression is reduced in diseased hearts from patients with ischemic heart failure and mice undergoing myocardial infarction surgery. This discovery reveals a novel crosstalk mechanism between mitochondrial and cytosolic translation processes, mediated by FAM210A and regulating mitochondrial translation elongation. Ribo-seq proved that mitochondrial translation elongation is compromised upon genetic knockout of *Fam210a* in cardiomyocytes in mice, as indicated by increased ribosome footprints of mRNAs transcribed from mitochondrial-encoded genes. Adeno-associated virus (AAV9)-mediated overexpression of FAM210A can significantly enhance mitochondrial translation and protect the heart from ischemia-induced cardiac dysfunction [39], making it a potential candidate for gene therapy (Figure 4). As a future direction, a hypermorphic mouse model with reduced FAM210A expression, such as a homozygous knock-in of a human mutation (if conserved in mice) or a heterozygous knockout, is more suitable for recapitulating the loss-of-function human mutation or decreased expression in myocardial infarction, thereby confirming the causal effects of decreased FAM210A level in heart diseases.

Following a prior miRNA expression screen for MI models [86], we identified mammalian miR-574 (including guide and passenger strands miR-574-5p and miR-574-3p) as involved in cardiac hypertrophy and pathological remodeling. We discovered that dual-strand miRNAs, miR-574-5p and miR-574-3p, are induced in human and mouse non-ischemic failing hearts compared to healthy hearts. Using the miR-574 genetic knockout mouse model and RNA-seq, we found that miR-574-5p and miR-574-3p target *Fam210a* mRNA in mouse cardiomyocytes and cardiac fibroblasts, thereby maintaining mitochondrial homeostasis and preventing cardiac hypertrophy and ventricular remodeling in a pressure overload-induced hypertensive cardiomyopathy model [87]. This work demonstrates that the miR-574-FAM210A axis regulates mitochondrial translation to maintain optimal expression of mitochondrial electron transport chain complex genes in non-ischemic heart disease [87] (Figure 4). More importantly, miR-574 delivered in the hypertensive cardiomyopathy mouse models via nanoparticles can be a potential therapeutic tool for antagonizing cardiac pathological remodeling [87]. Based on these findings, we proposed “normalizing” mitochondrial translation to maintain the homeostatic balance with cytosolic translation to protect the heart from progressive pathological remodeling and heart failure. In the future, cardiac cell type-specific knockout and transgenic overexpression of miR-574 can be used to deeply characterize the physiological and pathological functions in specific cell types and the causal relationship between miR-574 and heart disease.

To characterize the biochemical and biophysical properties, the protein structure of FAM210A and its complex with a mitochondrial translation elongation factor, EF-Tu, or other interacting partners needs to be resolved. This was highly challenging as FAM210A is a mitochondrial transmembrane protein. We overexpressed human FAM210A with a truncated mitochondria-targeting signal peptide at the N-terminus in bacteria and purified the recombinant protein from *E. coli* [88]. Interestingly, bacteria-derived translation elongation factor EF-Tu is co-purified with human FAM210A, which recapitulates the formation of the FAM210A-EF-Tu complex in cardiac mitochondria, as seen by immunoprecipitation-mass spectrometry [87]. Consistently, recombinant human FAM210A protein is localized in the plasma membrane of *E. coli,* like the localization in the inner mitochondrial membrane in CMs, indicating a late evolutionary event of introducing FAM210A as a regulatory factor for mitochondrial translation in multicellular organisms from worms to humans

### 3.3. Translational Regulation of Cardiac Cell Proliferation and Differentiation

Congenital heart disease (CHD) is the leading cause of birth defect-related death and can lead to severe adult heart disease, such as heart failure. The severity of CHD highlights the importance of the normal developmental program of the cardiovascular system, which relies on precise spatial and temporal control of gene expression that encodes structural proteins, transcription factors, and cell cycle-related proteins. The gene expression programs of developing organs are established at both transcription and translation levels at the early stages of embryonic development. Transcriptional and epigenetic mechanisms have been extensively studied for initiating and orchestrating developmental time courses during organogenesis. It is well-established that multiple transcription factors and cell cycle-related proteins regulate the development of the cardiovascular system [89,90,91,92]. However, the regulatory mechanism upstream of these factors at the translational level remains largely unexplored. Translational regulation is crucial for determining the fate of human embryonic stem cells (hESCs) towards cardiac differentiation through a specific mRNA translation regulatory pathway directed by RBPMS (RNA-binding protein with multiple splicing) [93]. Under cardiac cell differentiation conditions, RBPMS is associated with actively translating ribosomes in hESCs to activate the translation of a specific cohort of key factors required for initiating a cardiac commitment program, such as the Wingless/Integrated (WNT) signaling. As a result, the loss-of-function of RBPMS profoundly impairs cardiac mesoderm specification, thereby leading to profound morphogenetic and tissue patterning defects in cultured human cardiac organoids. RBPMS acts in translational control via two separate and related molecular mechanisms, including the selective binding to the 3′-UTR of specific target mRNAs and the global promotion of translation initiation, thereby enhancing protein synthesis. RBPMS depletion leads to the inhibition of translation initiation, as indicated by the abnormal retention of the eIF3 complex and the drop-off of eIF5A on mRNAs, thereby blocking ribosome recruitment and elongation during protein synthesis.

**Figure 5:**
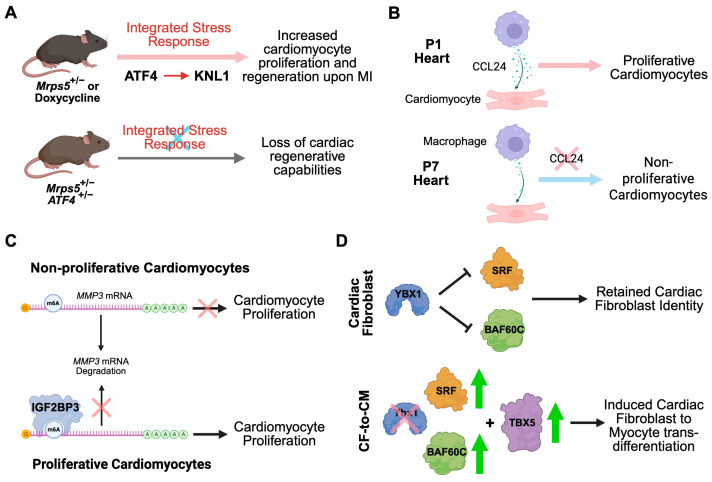
Translational regulatory mechanisms driving cardiomyocyte proliferation and cardiac fibroblast-to-myocyte transdifferentiation. (**A**). Mild mitochondrial translational defects activate integrated stress response and cardiomyocyte proliferation. (**B**). Paracrine secretion of CCL24 from macrophages stimulates cardiomyocyte proliferation. (**C**). The molecular mechanism of IGF2BP3-mediated binding and stabilization of m6A-modified *MMP3* mRNA contributes to cardiomyocyte proliferation. (**D**). Loss-of-function of YBX1 promotes cardiac fibroblast-to-myocyte trans-differentiation with TBX5 overexpression. Arrows with a crossed mark: the cause-and-effect relationship is lost. Green arrows: gene expression is increased.

Mitochondria play essential roles in maintaining normal cardiac function and preventing heart disease. Generally, reduced mitochondrial translation leads to a mitochondrial-nuclear proteomic imbalance and results in changes in the activity of the electron-transport chain complex. Intriguingly, deleting a single allele of mitochondrial small ribosomal subunit protein 5 (*Mrps5*) in mice enhances cardiomyocyte proliferation and cardiac regeneration in a surgical myocardial infarction mouse model [94] (Figure 5A). Cardiac function after MI surgery is significantly improved in mice with haploinsufficiency of *Mrps5*. Activating transcription factor 4 (ATF4) is a critical regulator of the mitochondrial stress response in cardiomyocytes from heterozygous *Mrps5* knockout mice. Mechanistically, ATF4 regulates KNL1 (kinetochore scaffold 1) expression, increasing cytokinesis during cardiomyocyte proliferation. Doxycycline-mediated inhibition of mitochondrial translation counterintuitively promoted cardiomyocyte proliferation. Cardiomyocyte proliferation of *Mrps5*^+/−^ can be attenuated when one allele of *Atf4* is also genetically deleted (*Mrps5*^+/−^ *Atf4*^+/−^), resulting in a loss of the cardiac regenerative capacity. MRPS5 reduction and doxycycline treatment activate an evolutionarily conserved regulatory mechanism that enhances the proliferation of human induced pluripotent stem cell (hiPSC)-derived cardiomyocytes, offering a novel approach for treating cardiac injury and promoting heart regeneration. An interesting clinical investigation following this animal study can be conducted to examine whether the heterozygote carriers of any nuclear-encoded mitochondrial gene nonsense mutations would be less susceptible to heart diseases compared to the general population without any loss-of-function mitochondrial gene mutations.

In addition to the stoichiometry of ribosomal proteins influencing cardiomyocyte proliferation, the posttranslational modifications contribute to cardiomyocyte differentiation. 2-oxoglutarate and iron-dependent oxygenase domain-containing protein 1 (OGFOD1), a ribosomal prolyl-hydroxylase, catalyzes the posttranslational hydroxylation of Pro^62^ in the small ribosomal protein S23 (RPS23). Genetic deletion of OGFOD1 in an in vitro cell culture model of human cardiomyocytes decreases the translation of specific proteins, such as RNA-binding proteins, which may, in turn, regulate translation and alternative splicing as a secondary effect [95]. Loss of OGFOD1 causes alterations in protein translation and reprograms the cardiac proteome, thereby increasing the synthesis of sarcomere proteins, including cardiac troponins, titin, and cardiac myosin-binding protein C. Consistent with these translational changes, OGFOD1 expression is reduced during cardiomyocyte differentiation.

Mammalian cardiomyocytes exit the cell cycle shortly after birth. The adult heart cannot regenerate in response to injury, whereas the neonatal heart can efficiently regenerate following MI surgery, but this capacity is lost by postnatal day (P)7 [96]. RNA-seq analysis for regenerative (P1) and nonregenerative (P8) mouse hearts after MI surgery revealed that the transcriptome of post-MI regenerative hearts reverts rapidly to a baseline pattern compared to uninjured control hearts. In contrast, post-MI nonregenerative hearts exhibited a distinct gene expression pattern [97]. Integrated with active chromatin landscapes, genes and biological processes activated in injured hearts were identified, among which the immune response and embryonic developmental gene programs were strikingly divergent between regenerative and nonregenerative hearts. The macrophage-mediated innate immune response has been reported to play a critical role in neonatal heart regeneration [98]. Acute activation of immune-related genes in regenerative hearts is evident through the deposition of histone H3 lysine-27 acetylation (H3K27ac), which marks active enhancers and promoters, serving as key steps in triggering regeneration; notably, the injury-induced immune factor CCL24 secreted from P1 macrophages promoted CM proliferation during neonatal heart regeneration (Figure 5B). Moreover, the regenerative P1 heart retained developmental and cell-cycle gene programs, within which an RNA-binding protein, IGF2BP3 (insulin-like growth factor 2 mRNA-binding protein), was identified to promote CM proliferation and restore cardiac morphology and function after MI, possibly driven by translational activation of CM regenerative factor mRNAs such as *IGF2* (insulin-like growth factor 2) among other target mRNAs through the binding of 3′-UTR.

Understanding the molecular mechanisms underlying the reactivation of CM proliferation and heart regeneration is essential for inducing cardiac repair in response to injury. IGF2BP3 belongs to a family of N6-methyladenosine (m^6^A) readers that recognize the consensus GG(m^6^A)C sequence and promote the stability of thousands of m^6^A-modified mRNA targets [99]. IGF2BP3 expression progressively declines in mouse hearts during postnatal development and is nearly undetectable by P28. While MI induces IGF2BP3 upregulation in neonatal hearts, its expression remains barely detectable in adult hearts [100]. Overexpression of IG2BP3 in P7 CMs promotes mitosis and cell-cycle progression, while IGF2BP3 knockdown decreases CM proliferation (Figure 5C). In vivo, adeno-associated virus AAV9-mediated IGF2BP3 overexpression in the left ventricular myocardium of P1 mice, followed by MI surgery, increases proliferative CMs and results in improved cardiac hypertrophy, reduced myocardial infarction size, decreased myocardial fibrosis, and preserved left ventricular ejection fraction. Among the most enriched mRNAs pulled down by IGF2BP3, *MMP3* mRNA was identified as a target of IGF2BP3-mediated post-translational regulation. Mechanistically, the KH3 and KH4 domains of IGF2BP3 directly bind to the *MMP3* mRNA, stabilizing it through m^6^A modification and thereby increasing MMP3 protein translation. Functionally, MMP3 acts as a downstream effector of IGF2BP3 to promote heart regeneration and improve cardiac function after myocardial infarction.

Cardiomyocytes lose their proliferative ability shortly after their differentiation and maturation. This does not allow adult mammalian hearts to regenerate after damage. Heart-specific triggers and pathways responsible for proliferation remain enigmatic. Understanding the mRNA expression signature in proliferating cardiomyocytes is a key to studying heart regeneration. A recent unbiased comparative study used artificial intelligence (AI)-based tools to find such signatures among two pre-existing in vivo (mice and pigs) and one in vitro (human induced pluripotent stem cell-to-cardiomyocyte, hiPSC-CM) proliferating CM model. In vivo mouse and pig models provide single-nucleus (sn)RNA-seq on different days after induced MI. MI was also performed on P28 in pigs alone or combined with another apical resection surgery on P1. The in vitro model includes bulk RNA-seq of the hiPSC-CM cells on different days after differentiation. The AI tool identifies clusters of proliferative cells based on RNA-seq analysis of hiPSC-CM 16 days after differentiation, when these cells maintain their proliferative ability, compared to hiPSC-CM 140 days after differentiation, when cells stop proliferating. Upregulated and downregulated mRNAs for each model and each cluster were found. Many upregulated genes are associated with mitochondrial metabolism, protein biosynthesis, and mRNA modification or processing. The investigators identified twenty-one overlapping up-regulated genes among all models across three species. Nine coded proteins are associated with ribosomes, including a well-established CM regenerative factor IGF2BP3, HSPA5, DHX9, and BLM, among others. Three genes code classic RNA-binding proteins (DHX9, PTBP3, and IGF2BP3); others are metabolic enzymes, cytoskeleton maintenance, and heat shock proteins, which have been identified as noncanonical RBPs [101]. Immunohistochemistry in hiPSC-CM proved overexpression of multiple proteins in pig hearts with proliferating CMs, such as PTBP3, DHX9, DDX6, and HNRNPUL1. The biological function and biochemical mechanism of these RNA-binding proteins involved in CM proliferation and heart regeneration in rodents and large mammals warrant further studies to provide novel tools for regenerative medicine.

In addition to hESC/hiPSC-CM differentiation and reactivating cardiomyocyte proliferation, direct reprogramming of cardiac fibroblasts into induced cardiomyocytes (iCMs) is another promising strategy for heart regeneration [102,103]. Recent studies have revealed new insights into the translational landscape underlying the CF-to-iCM trans-differentiation and reprogramming process through integrative translatomic and transcriptomic profiling [38]. A gene-specific targeted loss-of-function screening for translational regulatory factors identified an RNA-binding protein, Y-box binding protein 1 (YBX1), as a critical barrier to iCM trans-differentiation (Figure 5D). In a mouse myocardial infarction-induced heart failure model, reducing Ybx1 expression enhanced the efficiency of CF-to-iCM reprogramming in vivo, resulting in improved cardiac function and decreased fibrosis and scar size. *Ybx1* removal activates the translation of its direct mRNA targets *Srf* and *Baf60c*, which directs the effect of *Ybx1* depletion on facilitating iCM induction. Depletion of *Ybx1* combined with overexpression of a single transcription factor, *Tbx5,* is sufficient in mediating CF-to-iCM conversion. This strategy simplifies the well-established overexpression of the transcription factor “cocktail”, including GATA4, MEF2C, and TBX5, with a specific stoichiometric ratio [104].

## 4. Translational Control in Adult Cardiac Disease

### 4.1. Translational Control in Cardiomyocyte Hypertrophy

The human translation machinery is comprised of three main parts: ribosomes (ribosomal proteins: RPs; ribosome RNA [rRNA]), translation factors (initiation and elongation factors), and ARSs and substrate transfer RNAs (tRNAs) [105] (Figure 1). Elevated global mRNA translation and protein synthesis are common features in cardiac hypertrophy [106,107,108]. Hypertrophic stimuli activate mTOR (mammalian target of rapamycin) signaling [109,110,111], which promotes the synthesis of ribosomal proteins and the activation of translation factors [112]. In hypertrophic CMs, ribosomal proteins and rRNAs are markedly increased to support the increase of protein mass and CM size [106,113,114,115]. Therefore, both the capacity and efficiency of translation are increased in response to hypertrophic stimuli. Several specific translation factors required for cardiac hypertrophy have been identified.

#### 4.1.1. Role of Translation Initiation Factors in Cardiac Hypertrophy

Theoretically, translation efficiency can be regulated in three steps (i.e., initiation, elongation, and termination). In mammals, translation initiation is a critical control point in most cellular responses [116]. Translation initiation is the process in which the assembly of elongation-competent 80S ribosomes occurs at the initiation codon, allowing further elongation to synthesize a full-length protein. Generally, the initiation process comprises two steps: forming 48S initiation complexes and joining these 48S complexes with 60S large ribosomal subunits. Translation initiation happens canonically at the 5′ end of mRNAs harboring a 7-methylguanosine (m^7^G) cap, i.e., cap-dependent translation initiation, which requires the cooperation of multiple translation initiation factors [116,117] (Figure 1 and Figure 3). Alterations in translation initiation are a crucial feature of cancer, viral infections, and cardiac hypertrophy [118,119,120].

**Figure 6:**
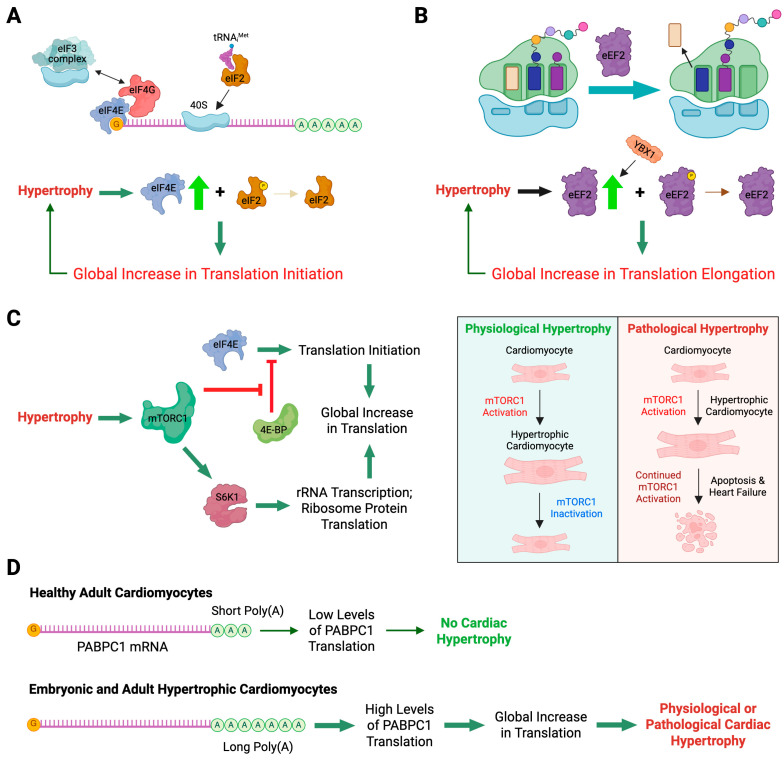
Translational control mechanisms in cardiomyocyte hypertrophy. (**A**) Increased expression of eukaryotic translation initiation factor 4E (eIF4E) promotes global translation to support cardiomyocyte hypertrophy. (**B**) Increased expression of eukaryotic translation elongation factor 2 (eEF2) enhances global translation to promote cardiac hypertrophy. (**C**) Activation of mTORC1 kinase activity phosphorylates 4EBP1 and S6K1 and enhances eIF4E-mediated cap-dependent translation initiation, rRNA transcription, and ribosome biogenesis, thereby enhancing global mRNA translation and cardiomyocyte hypertrophy. (**D**) Poly(A)-binding protein (PABPC1) upregulation increases global mRNA translation and cardiac hypertrophy.

The changes in protein synthesis rate in cardiomyocytes during stress-induced cardiac hypertrophy have been shown to correlate with changes in the activity of translation initiation factors, which modulate the rate of translation initiation [121,122,123]. For instance, the activity of translation initiation factor eIF4E is increased upon hypertrophic stimuli in cardiomyocytes in a pressure overload model [120,124] (Figure 6A). eIF4E is one component of the eIF4F complex that interacts with the m^7^G cap. When activated, eIF4E facilitates the loading of the 43S initiation complex onto mRNA to form 48S initiation complexes and promotes ribosome scanning in the 5′-UTR. The activity of eIF4E depends on its phosphorylation [125] (Figure 3). Expression of eIF3E has been shown to increase during both electrical pacing- or α1-adrenoceptor-induced hypertrophy of quiescent neonatal rat CMs [124]. Phosphorylation of eIF4E is increased in adult feline CMs in culture during electrical pacing and in canine CMs in vivo after imposition of pressure overload [120]. An increase in translation efficiency was concomitant with an increase in eIF4E phosphorylation in hypertrophied CMs in vivo [123]. Overexpression of an inactive form of eIF4E slows down protein synthesis and reduces CM hypertrophy [126]. However, increased expression or phosphorylation of eIF4E alone cannot induce hypertrophy in non-stimulated CMs [126]. Thus, increased eIF4E activity is required for the accelerated rate of protein synthesis following pressure overload; however, increased eIF4E activity alone is insufficient for hypertrophy, indicating the necessity of an increased stoichiometrically matched eIF4E-eIF4G1-eIF3 complex.

Besides eIF4E, modulated eIF2 activity has also been found after hypertrophic stimuli and other cardiovascular stress conditions [83,127,128]. eIF2 is essential for forming the eIF2-GTP-Met-tRNA^Met^ ternary complex during translation initiation. It cycles between GTP-bound active and GDP-bound inactive forms through a process of phosphorylation. Phosphorylation of eIF2 locks eIF2 in the GDP-bound inactive form, reducing translation initiation [129]. Glycogen synthase kinase 3b (GSK-3β), a kinase for eIF2Bε-S535 phosphorylation, is inhibited in isoproterenol-induced hypertrophied neonatal rat CMs, resulting in decreased eIF2 phosphorylation and accelerated translation initiation. Decreases in eIF2Bε-S535 phosphorylation were also observed in a rat model of cardiac hypertrophy in vivo [127]. Overexpression of a consistently phosphor-active eIF2Bε mutant causes hypertrophic growth, similar to that induced by isoproterenol treatment. In contrast, a phosphor-inactive eIF2Bε mutant blocks the effect of isoproterenol treatment [127]. Therefore, unlike eIF4E, activated eIF2 alone is sufficient to cause CM hypertrophy (Figure 6B). Furthermore, the modulation of eIF2α phosphorylation has also been found to be related to cardiovascular stress [83,128]. *Gcn2*^−/−^ mice lacking a key eIF2α kinase were less prone to ventricular dysfunction, myocardial apoptosis, and fibrosis when subjected to transverse aortic constriction [128]. A dephosphorylation inhibitor, salubrinal, has been shown to attenuate pressure-overload-induced cardiac hypertrophy [130]. Although modulation of translation initiation factor activity correlates with cardiac disease, the global effect and high conservation of these factors among different cell types make it difficult to propose a treatment specific to cardiac disease without affecting other organ functions or essential biological processes.

In addition to the translation initiation rate, the fidelity of translation initiation is also associated with cardiac function and disease [67]. An example of this is mitochondrial translation, which synthesizes 13 essential proteins for the electron transport chain complex to assemble the oxidative phosphorylation (OXPHOS) system required for energy production. One mitochondrial translation initiation factor, MTIF3, is required for molecular proofreading during the mitochondrial translation process. Loss of MTIF3 will increase the protein synthesis rate at the expense of reduced translation initiation fidelity, resulting in uncoordinated translation of the electron transport chain complex proteins, reducing the correct assembly of the OXPHOS system and ATP production [67]. Heart-specific depletion of MTIF3 in mice causes spontaneous dilated cardiomyopathy, likely due to mitochondrial dysfunction in cardiac cells [67].

Apart from the canonical cap-dependent initiation, translation initiation can also occur in an alternative manner at internal ribosome entry sites (IRES) [131,132]. Cap-independent translation initiation is associated with many diseases, including cardiac diseases [131,133,134]. One example of an IRES is Connexin 43 (Cx43) and its truncated isoform GJA1-20k [135,136]. Cx43 is the most widely expressed gap junction protein translated from an IRES in the 5′ uORF [137], while GJA1-20k is a truncated protein isoform generated from an IRES within the mORF of Cx43 [136]. Levels of GJA1-20k regulate the formation of Cx43 gap junctions, which in cardiomyocytes are necessary for the electrical conduction that facilitates heartbeat [133]. The IRES activity of GJA1-20k is increased in response to hypoxic conditions in cardiomyocytes [136]. Ectopic expression of GJA1-20k has been found to rescue gap junction loss during acute ischemia, proving that modulating alternative translation initiation may protect against loss of electrical coupling, particularly in heart disease [133]. The translation of GJA1-20k may be more complicated than “normal” IRES-driven translation, as evidence suggests that m^7^G cap and ribosome scanning are also required [138]. The non-canonical translation initiation of GJA1-20k exemplifies the role of alternative translation initiation related to cardiac disease. In the future, the mechanism needs to be elucidated regarding the regulation of the stoichiometric ratio of GJA1-20k to full-length GJA1 at the translational level under both baseline and cardiac stress conditions.

#### 4.1.2. Role of Translation Elongation Factors in Cardiac Hypertrophy

Although translation initiation has been established as the primary step for translational regulation [116], it is not surprising that translation elongation is also highly regulated since it is the most energy-consuming step during protein synthesis [139]. Translation elongation is the process by which ribosomes decode mRNAs by bringing in the proper amino acids carried by aminoacyl-transfer RNAs (aa-tRNA) to form peptides (Figure 1). This process includes the selection of aa-tRNA according to the mRNA codon in the ribosome’s aminoacyl (A) site, peptide bond formation, and movement of both tRNAs and the mRNA through the ribosome [140]. A complex set of factors, either individually or cooperatively, affects either the rate or the precision of translation elongation [139]. The translation elongation step is affected by modification, abundance, and aminoacylation status of tRNAs, as well as the activity of elongation factors [139]. Understanding the regulation of translation elongation is essential, as it has been established that dysregulation of elongation is related to various human diseases (e.g., heart disease [106], tumors [141], neurodegeneration [142]). Evidence of the correlation between the regulation of translation elongation and cardiac disease is evident in changes to the phosphorylation status of the eukaryotic elongation factor 2 (eEF2) induced by hypertrophic stimuli [143,144,145,146] (Figure 6B). eEF2 is a GTPase catalyzing the GTP-dependent ribosome translocation step during translation elongation. eEF2 enters the A-site and induces a conformational change of the ribosome through GTP hydrolysis, allowing the newly formed A-site-bound peptidyl-tRNA and P-site-bound deacylated tRNA to move to the P and E sites. The ribosome can then recruit a new aminoacyl-tRNA into the A-site and continue peptide elongation [147]. The activity of eEF2 largely depends on its phosphorylation status [147]. Dephosphorylated eEF2 results in eEF2 activation and increases elongation rate, while phosphorylation of eEF2 at Thr^56^ blocks translation elongation [144]. The growth rate of CMs also correlates closely with eEF2 phosphorylation [143]. Hypertrophic growth of neonatal CMs induced by angiotensin II is associated with mitogen-activated protein kinase-dependent decreases in eEF2 phosphorylation [144]. Decreased eEF2 phosphorylation can be detected within 30 min after angiotensin II treatment in isolated neonatal CMs, together with increased protein synthesis, which is believed to be one of the primary causes of cardiac hypertrophy [144]. Other hypertrophic agonists, including endothelin-1, phenylephrine, and insulin, are also reported to facilitate the dephosphorylation of eEF2 in CMs [145], suggesting that hypertrophy correlates with increased translation elongation caused by eEF2 dephosphorylation. However, in another model where isoproterenol was used to induce hypertrophy in isolated adult rat ventricular myocytes, increased eEF2 phosphorylation was observed as early as 1 min after isoproterenol treatment and remained elevated for 20 min [146]. The inconsistent results of different models suggest that modulation of translation elongation during hypertrophy is a complex and dynamic process. Isolated pharmaceutical hypertrophy models in vitro may not reflect what happens in vivo. In vivo mouse studies have shown that changes in eEF2 phosphorylation remain undetectable 1 week after isoproterenol injection into adult mice, while eEF2 phosphorylation is significantly increased after transverse aortic constriction surgery [143].

Another example illustrating the correlation between elongation regulation and hypertrophy comes from eukaryotic elongation factor 1A (eEF1A) [30]. eEF1A promotes the GTP-dependent binding of aminoacyl-tRNA into the A-site of ribosomes, allowing downstream interactions to add one amino acid to the elongating peptide. The activity of eEF1A depends on its essential cofactor, eEF1B2 [148]. TIP30 has been identified as an endogenous translation elongation inhibitor that interacts with eEF1A to prevent its association with eEF1B2. *Tip30*^+/−^ and *Tip30*^−/−^ mice are prone to developing cardiac hypertrophy and heart failure after transverse aortic constriction surgery. Overexpression of Tip30 in neonatal rat CMs restricts hypertrophy induced by hypertrophic growth factors, phenylephrine, or endothelin-1 [30].

Besides cytosolic translation elongation, mitochondrial translation elongation is highly regulated and relevant to cardiac disease. One example of this is the correlation between mitochondrial translation and ischemia [149]. Mitochondrial dysfunction is a well-characterized feature of ischemic myocardial injury [150]. Translation in the mitochondria produces 13 peptides essential for the electron transport chain and OXPHOS [151]. Ischemic stimuli partially affect OXPHOS and energy production by regulating mitochondrial translation elongation. Mechanistically, ischemia increases the phosphorylation of the mitochondrial translational elongation factor Tu (EF-Tu) [149]. Unphosphorylated EF-Tu forms a ternary complex with GTP and mitochondrial aminoacyl-tRNAs, facilitating the recruitment of aminoacyl-tRNAs to the A-site of the elongating ribosomes [151]. Phosphorylation on Thr^382^ of EF-Tu inhibits this process by preventing ternary complex formation, thus inhibiting protein synthesis [152]. Increased phosphorylation of EF-Tu has been observed in mouse hearts subjected to ischemia followed by reperfusion [149]. Incubation of isolated non-ischemic mitochondria in cytosol from ischemic hearts increases EF-Tu phosphorylation. In contrast, incubation of mitochondria in cytosol from healthy hearts decreases EF-Tu phosphorylation compared with the absence of cytosol, suggesting that a mechanism in the cytosol to prevent EF-Tu phosphorylation and maintain the normal function of mitochondria is inhibited under ischemic conditions [149].

Apart from elongation factors, the rate of protein synthesis is also regulated by the availability of ribosomes, which affects the capacity of translation elongation [106,139]. It has been demonstrated that increased ribosome content is required for the hypertrophic growth of CMs under phenylephrine stimulation [153]. Increased transcription of the 45S rDNA (encoding rRNA for ribosome assembly) has been observed in cardiac hypertrophy induced by phenylephrine, endothelin-1, phorbol myristate acetate, angiotensin II, and contraction, partially through an increase in RNA polymerase I activity [114,115,154]. Ribosomal proteins are also increased to coordinate with the increased rRNAs through enhanced translation. S6K1, a mitogen-stimulated protein kinase downstream of mTOR, has been shown to account for the increased translation of ribosomal proteins [155]. S6K1 phosphorylates and activates the 40S ribosomal protein S6, facilitating the translation of multiple ribosomal protein mRNAs harboring a 5′ polypyrimidine tract [155]. Phosphoinositide 3 kinase (PI3K), upstream of S6K1 but downstream of mTOR, has also been shown to promote hypertrophic growth of CMs in a transgenic mouse model [156].

#### 4.1.3. Genetic Loss-of-Function of mTORC1 Causes Heart Failure in Mice

The mammalian target of rapamycin C1 (mTORC1) is a master regulator of cell growth and survival. Activation of mTORC1 induces protein synthesis in response to physiological and pathological stimuli via increased cellular translational activity. Notable dysregulation of translational events indicates the acute cellular response incurred during cardiac stress and remodeling, which ultimately culminates in cardiac diseases or heart failure if it persists [41,157].

For mTORC1-mediated translational initiation, mTORC1 activation induces the phosphorylation of its major downstream effectors, including ribosomal S6 kinase 1 (S6K1) and eIF4E-binding protein-1 (4E-BP1) [158]. S6K1 is required in ribosome biogenesis primarily via positive regulation of pyrimidine biosynthesis and rDNA transcription. Thus, mTORC1 activation through S6K1 can direct rRNA production to synthesize more ribosomes, thereby impacting protein translational activity [159,160,161]. Whereas 4E-BP1 can bind to the cap-binding protein eIF4E and prevent it from interacting with other translation initiation factors to form the eIF4F complex, which is essential during the formation of the 48S initiation complex in translation initiation [162]. In other words, mTORC1 activation increases phosphorylated 4E-BP1, which relieves the inhibitory constraint on eIF4E, culminating in augmented translation initiation.

mTORC1 is essential in normal cardiac physiology (Figure 6C). mTORC1 plays a crucial role in CM proliferation and growth during murine heart development [163,164] despite its initial low expression in the heart at embryonic stages. In adult mice, mTORC1 deficiency mediated by inducible cardiac-specific *Raptor* and *Mtor* ablation leads to lethal dilated cardiomyopathy. *Raptor* ablation in the myocardium results in severe heart failure within 6 weeks of gene deletion. Re-expression of the fetal gene program precedes the cardiac phenotypes, indicating that the cardiac stress response is initiated by the overexpression of ANP, BNP, and β-MHC in the myocardium [165]. *Mtor-*ablated myocardium demonstrates enhanced apoptosis and impaired mitochondrial function [166]. Thus, a negative causal relationship is evident between mTORC1 loss-of-function and cardiac dysfunction. Under pathological stimuli, mTORC1 deficiency impairs the myocardial response to cardiac stress at postnatal stages. Upon pressure overload, reduced mTORC1 activity in *Mtor* ablated mice hampers the hypertrophic response and accelerates heart failure progression [166]. Compared to wild-type mice, *Raptor-*ablated mice show no cardiac hypertrophy at the organ or tissue level after aortic constriction [165]. This supports the hypothesis that mTORC1 inactivation attenuates the translation activity required to induce an adaptive hypertrophic response, thereby preserving cardiac function and integrity upon pressure overload or hemodynamic stress. However, the adaptive hypertrophic response, which is beneficial to cardiac function and requires mTORC1, converts to pathological hypertrophy if the pathological stimuli are sustained. Therefore, selective and partial inhibition of mTORC1 is cardioprotective in response to persistent pathological hypertrophy [157,167,168,169]. Rapamycin treatment attenuates load-induced cardiac hypertrophy, associated with reduced left ventricular diastolic and systolic diameters, and improved cardiac contractile function [167,168]. *Rheb1* deletion inhibits mTORC1 activation, conferring a cardio-protective effect in myocardial infarction and transverse aortic constriction mouse models [169]. Furthermore, overexpression of PRAS40, an mTORC1 inhibitor, suppresses pathological hypertrophic growth in CMs. PRAS40 gain-of-function mice exhibited decreased heart size, reduced perivascular fibrosis, and preserved systolic function compared to control mice under cardiac stresses. mTORC1 inhibition, mediated by PRAS40 overexpression or rapamycin treatment, can effectively regress the established pathological remodeling and hypertrophy in mice with pressure overload [157,168]. Collectively, mTORC1 inhibition may serve as a therapeutic strategy to prevent heart failure progression, and rapamycin is currently tested in clinical trials for the treatment of HFpEF (Figure 6C).

In conclusion, mTORC1 plays a multifaceted role in heart development and disease. Complete genetic inactivation of the mTORC1 signaling is detrimental to maintaining normal cardiac physiological function, thereby leading to cardiac pathological remodeling and heart failure. In contrast, partial and selective inhibition of the mTORC1 pathway holds promise as a potential therapeutic strategy in certain cardiac pathologies by mitigating maladaptive responses while preserving essential physiological functions. Further clinical studies are crucial to understand the intricate pathophysiological effectsof this pathway and to guide the development of safe and effective treatments for various heart diseases.

#### 4.1.4. PABPC1-Mediated Translational Control of Physiological and Pathological Cardiac Hypertrophy

Poly(A)-binding protein C1 (PABPC1) interacts with the 3′ poly(A) tail of mRNAs and the eukaryotic translation initiation factor 4G (eIF4G) to promote mRNA circularization and translation initiation efficiency. PABPC1 protein expression is high in the embryonic heart but becomes undetectable in the adult heart, with no difference at the level of *Pabpc1* mRNA in mice [107] (Figure 6D). The *Pabpc1* mRNA has a substantially shorter poly(A) tail in the adult heart, leading to extremely low translation efficiency. In contrast, the poly(A) tail length on the *Pabpc1* mRNA and the expression level of the PABPC1 protein are restored in adult hearts during cardiac hypertrophy. This mechanism is conserved in both exercise-triggered physiological hypertrophy and heart disease-caused pathological hypertrophy, with the common feature of enhanced protein production in cardiomyocytes. Transgenic overexpression of PABPC1 in adult hearts is sufficient to drive global protein synthesis and cardiac hypertrophy in mice [107]. Future studies can measure the poly(A) length of *PABPC1* mRNA and other mRNAs across the transcriptome in hearts of heart failure patients and non-failing normal humans to generalize this polyadenylation mechanism to other transcripts in humans.

#### 4.1.5. Translational Control of Ybx1 Expression Regulates Cardiac Function in Response to Pressure Overload In Vivo

Y box-binding protein 1 (YBX1), an RNA-binding protein, is known to regulate translation by binding to target mRNAs. In the hearts undergoing pathological remodeling, the mTORC1 signaling pathway activates the translation of *Ybx1* mRNA without altering its transcription level [13]. RNA immunoprecipitation (RIP)-seq uncovered *Eef2* (eukaryotic translation elongation factor 2) mRNA as a critical target of YBX1. *Eef2* mRNA contains a mTORC1-regulated terminal oligopyrimidine motif in the 5′-UTR. eEF2 protein, upregulated during cardiac pathological remodeling, enhances global protein synthesis and promotes cardiac hypertrophy. AAV9-mediated expression of *Ybx1* shRNA reduced cardiomyocyte hypertrophy and restored left ventricular contractile function. Therefore, YBX1 is considered a promising therapeutic target, as inhibiting or reducing YBX1 expression in cardiac fibroblasts and myocytes compromises both cardiac fibrosis [38] and hypertrophy [13]. It remains unclear whether the knockout of YBX1 in cardiomyocytes triggers any spontaneous cardiac phenotypes in animal models at baseline, despite the potential genetic redundancy of YBX1 with two other family members, YBX2 and YBX3, as YBX3 is also highly expressed in cardiomyocytes and other non-myocyte cardiac cells in humans according to the ProteinAtlas databases.

#### 4.1.6. m^6^A-Dependent Translational Control in Maintaining Normal Cardiac Function

Chemical modification of N6-methyladenosine (m^6^A) in mRNAs regulates cardiomyocyte function by controlling mRNA stability or translatability. Increased global m^6^A levels have been identified as a shared feature in heart failure with different etiologies. m^6^A reader proteins orchestrate gene expression in cardiac pathological remodeling. For instance, a key m^6^A reader protein, YTHDF2, controls cardiac function. Genetic deletion of *Ythdf2* in cardiomyocytes of mice causes mild cardiac hypertrophy, increased fibrosis, and reduced heart function in response to pressure overload stress and during cardiac aging [170]. The eukaryotic elongation factor 2 (eEF2) is post-transcriptionally regulated by YTHDF2 as indicated by cell-type-specific ribosome profiling analysis. Another study identifies that m^6^A methylation is directly associated with the progression of heart failure in both humans and mice. In this study, they identified a transcription-independent mechanism of mRNA translation regulation, where alterations in m^6^A methylation on mRNAs result in differential polysome occupancy and changes in protein expression. Furthermore, RNA demethylase *Fto* knockout in cardiomyocytes of mice shows impaired heart function, suggesting that mRNA m^6^A methylation is a potential target for therapeutic interventions [171].

### 4.2. Translational Control in Cardiac Fibroblast Activation During Fibrosis

#### 4.2.1. Translational Regulation in Human TGFβ-Activated Cardiac Fibroblasts

Fibrosis is a substantial symptom in multiple types of heart diseases and many other organ disorders. It can occur in inherited cardiomyopathy, ischemic heart disease, HFpEF, diabetes, and aging [172,173]. Cardiac fibrosis is primarily caused by excessive extracellular matrix accumulation secreted by highly specialized myofibroblast cells [173]. Myofibroblasts are generated through a process called cardiac fibroblast-to-myofibroblast transformation, wherein quiescent fibroblasts in the myocardium transdifferentiate into active myofibroblasts upon profibrotic stimuli such as transforming growth factor β (TGFβ), angiotensin II, endothelin-1, or serum response factor [173]. TGFβ has been identified as a primary and potent mediator of myofibroblast transformation. The induction of myofibroblast transformation by TGFβ appears to be a universal phenomenon across different organs [8,173,174]. Expression of TGFβ is upregulated upon injury, and the protein is subsequently secreted into the ECM, where it binds to either type I or type II TGFβ receptors on the surface of cardiac fibroblasts [173,174]. The promotion of myofibroblast transformation by TGFβ primarily depends on the activation of profibrotic gene transcription either through phosphorylation of transcription factor SMAD2/3 (canonical signaling) or mitogen-activated protein kinase (MAPK) signaling (non-canonical signaling) [173]. The significant downstream effector of TGFβ, interleukin-11 (IL-11), is related to the translational regulation of profibrotic gene expression required for myofibroblast transformation [8].

IL-11 is a hematopoietic IL-6 family cytokine with pleiotropic effects. Obana, M. et al. first demonstrated its correlation with cardiac fibrosis by showing that IL-11 was upregulated at the mRNA level in a mouse model 1 day after MI [175]. They also suggested that administering recombinant human IL-11 to mice after an MI operation, reduced cardiac fibrosis on day 14 [175]. However, Schafer S. et al. strongly challenged this opinion, suggesting mice cannot respond to human IL-11 due to species specificity of the ligand-receptor pairs [8]. In contrast, Schafer S. et al. suggested that IL-11 contributes to cardiac fibrosis downstream of TGFβ. In their studies, IL-11 increased the expression of profibrotic proteins without changing the mRNA level, indicating translational or at least posttranscriptional regulation. IL-11 was also knocked down or overexpressed in a mouse model undergoing MI to show its induction of fibrosis. However, they did not elucidate the mechanism by which profibrotic protein translation is induced by IL-11. Although there are still arguments supporting the inhibiting effect of IL-11 on fibrosis, IL-11 is more widely accepted as a contributor to cardiac fibrosis and other organ aging processes, with the potential to become a drug target, according to recent pre-clinical studies [8,176].

Since human TGFβ plays a pleiotropic role in inducing various pathological conditions, unraveling the key downstream regulator driving the pathogenesis of cardiac fibrosis is important. A comprehensive computational study [177] using RNA-seq and Ribo-seq databases of human heart samples is consistent with previous observations [35], wherein the incurred cardiac fibrotic response is reflected by the modulation of approximately one-third of all genes at the translational level. Hence, understanding the molecular mechanism underlying the activation of fibroblasts into myofibroblasts is of great interest for the development of more effective and specific anti-fibrotic therapies.

During pro-fibrotic response induced by TGFβ in primary human atrial cardiac fibroblasts, ribosome profiling reveals 1691 differentially translated mRNAs [41]. Specifically, the translating ribosome is augmented in mRNAs of ferritin light chain (*FTL*) and ferritin heavy chain 1 (*FTH1*), while it is less pronounced in integrin subunit alpha 3 (*ITGA3*), although the transcript abundance remains unchanged. Intriguingly, the effect of the pro-fibrotic response on translational dynamics is the most prominent at 24 h despite a gradual decrease at 2 and 6 h after TGFβ induction. Differentially transcribed genes and translated mRNAs imply that the profibrotic response induced by TGFβ alters mRNA abundance and translation efficiency differently. Hence, these can be further categorized into several distinct gene expression regulations: forwarded, exclusive, buffered, and intensified. Forwarded genes exhibit unchanged ribosome occupancy, despite no alteration in mRNA levels. Exclusive genes have altered ribosome occupancy but unchanged mRNA levels. Both ribosome occupancy and mRNA levels are modified in buffered and intensified genes. For example, gene expression regulation of transcription factor *HES1* is known as an intensified gene due to even more robust protein upregulation than would be expected by qPCR or RNA sequencing data alone. The upregulation effect at the translational level culminates in a prominent increase in the HES1 protein level, exceeding the transcriptional surge.

#### 4.2.2. EPRS1 Promotes Cardiac Fibrosis by Enhancing Proline-Rich Extracellular Matrix Protein Translation

Cytoplasmic glutamyl-prolyl-tRNA synthetase (EPRS1), the only bi-functional ARS in higher eukaryotic organisms (from C. elegans to humans), catalyzes the attachment of two amino acids, glutamic acid (E) and proline (P), to their cognate tRNAs for protein synthesis. In addition to EPRS1’s canonical aminoacylation function in translation, our previous work and others’ studies have shown that EPRS1 exerts a noncanonical function in translational silencing of inflammation-related mRNAs (e.g., *VEGFA*) in monocytes or macrophages as an anti-inflammatory response [12,21,178,179,180]. Hypoactive mutations in the PRS (prolyl-tRNA synthetase) domain of EPRS1 lead to hypomyelinating leukodystrophy [181,182] without causing cardiac dysfunction in patients, implying that reduced PRS enzymatic activity does not adversely affect the heart.

**Figure 7:**
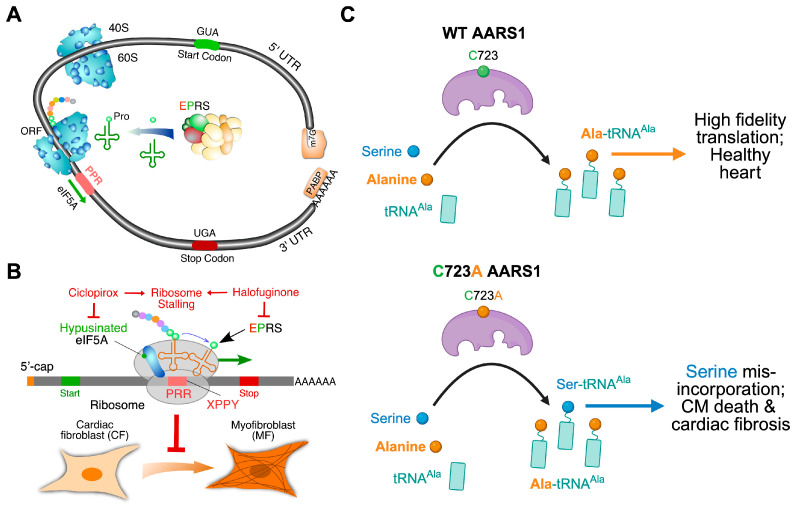
The mechanisms of dysfunctional aminoacyl-tRNA synthetases in cardiac fibrosis. (**A**) Increased glutamyl-prolyl-tRNA synthetase (EPRS1) and hyperactive eIF5A are essential for proline-rich pro-fibrotic extracellular matrix protein translation elongation. (**B**) (E)PRS1 inhibitor halofuginone and eIF5A hypusination inhibitor ciclopirox reduce the translation of proline codon-rich mRNAs encoding profibrotic proteins and inhibit CF-to-MF trans-differentiation. (**C**) Editing domain-inactivating mutation C723A in alanyl-tRNA synthetase (AARS1) causes mis-incorporation of incorrect serine amino acid to alanine codons in the proteome in the cardiac muscle, leading to severe cardiac fibrosis.

The role of cardiac EPRS1 in the heart has been recently elucidated. EPRS1 is the sole common ARS gene, containing single-nucleotide polymorphisms (SNPs) associated with congenital heart disease in humans [183], and found in TGFβ-induced ARSs in human cardiac fibroblasts [8], mouse GWAS of isoproterenol-induced cardiomyopathy model [184], and isoproterenol-induced ARSs in mouse failing hearts [185]. Our work shows that EPRS1 is induced in cardiac fibrosis triggered by multiple pro-fibrotic and pro-hypertrophic stimuli (e.g., isoproterenol, TGFβ, IL-11) as an integrated node for translational control in human and mouse cardiac remodeling and fibrosis [31]. EPRS1 activation contributes to elevated translation of proline-rich mRNAs via enhanced translation elongation in cardiac fibroblasts (Figure 7A).

EPRS1 expression is correlated with the progression of cardiac fibrosis [31]. Elevated EPRS1 expression was observed in human hearts with dilated cardiomyopathy and ischemic heart failure. A similar elevation in EPRS1 was observed in multiple HF mouse models, including isoproterenol-driven neurohormonal stimulation, transverse aortic constriction-induced pressure overload, and myocardial infarction with ischemic and inflammatory stresses. A reduced EPRS1 level in haploinsufficient (*Eprs1*^+/−^) mice and tamoxifen-inducible, myofibroblast-specific *Eprs1* conditional knockout mice reduces cardiac fibrosis and partially restores left ventricular contractile function in vivo. However, ribosome profiling has not been performed to map ribosome stalling or collision sites at the transcriptome-wide level and needs to be conducted in the future to fully understand how EPRS preferentially facilitates the translation of mRNAs containing proline-rich codons, among other features. Using the PRS-specific inhibitor halofuginone, EPRS1 was shown to be essential for the translation of many proline-rich mRNAs in cardiac fibroblasts, including many collagen proteins and secretory signaling proteins (e.g, LTBP2 and SULF1), among other profibrotic ligands, receptors, and enzymes (Figure 7B). These genes are considered novel anti-fibrotic targets for developing therapeutic approaches to treat heart failure with severe cardiac fibrosis. Interestingly, multiple pro-fibrotic (e.g., TGFβ and IL-11) and pro-hypertrophic agents (e.g., isoproterenol and angiotensin II) induce transcription of *EPRS1* mRNA. Possibly, extracellular signals activate EPRS1 expression, promoting the expression of pro-fibrotic proteins and cardiac scar formation. As a forward feedback loop, increased EPRS1 can promote multiple key pro-fibrotic protein translation, such as IL-11 [186], that further amplifies fibrogenic responses. The reduced level of charged Pro-tRNA^Pro^ following the reduction of EPRS1 activity or amount might, in turn, cause ribosome stalling and collisions on proline-rich codons and enhance no-go mRNA decay due to reduced ribosome occupancy [187]. Although EPRS1 is ubiquitous in all cells, proline-rich transcripts will likely vary in different cell types. Thus, halofuginone is likely to cause mRNA-selective and cell-type-specific translational repression, dependent on the heterogeneity of the proline-rich proteome across distinct cell types, such as cardiomyocytes [188].

#### 4.2.3. eIF5A: An Anti-Fibrosis Target Translation Elongation Factor

The biosynthesis of poly-proline peptide-containing proteins tends to cause ribosome stalling and translation arrest because of slow peptide bond formation [189,190]. A specialized translation elongation factor P (EF-P) overcomes this ribosome stalling effect in bacteria [189]. As a homolog of EF-P, eIF5A (eukaryotic initiation factor 5A, it is actually “elongation factor 5, EF5”) binds to the stalled ribosome between the peptidyl-tRNA binding and the tRNA-exiting sites, and facilitates peptidyl-transferase activity, thus resuming translation at poly-proline codons among other proline-rich codon motifs [190]. Eleven proline-proline dipeptide-containing motifs (XPPY) are among the top motifs for eIF5A-mediated ribosome readthrough in eukaryotic cells [190]. Hypusination of an evolutionarily conserved lysine residue in EF-P or eIF5A is essential for its biological function [191]. eIF5A inhibitors have also been tested in pre-clinical models for treating cancer and metabolic syndrome [192,193]. eIF5A may serve as a novel anti-fibrotic therapeutic target, and eIF5A inhibitors can be used to inhibit organ fibrosis in the future. For example, an antibiotic, ciclopirox, was repurposed to reduce cardiac fibrosis by inhibiting eIF5A activity in animal models of heart failure, following myocardial infarction [194] (Figure 7B). In the future, the role of eIF5A, its hypusination-dependent activity, and upstream catalytic enzymes need to be thoroughly investigated in cardiomyocytes to better understand the cardioprotective effect of spermidine, a precursor metabolite for hypusination of eIF5A in humans [195], as well as the impact of hypusinated eIF5A in heart failure with reduced or preserved ejection fraction as both manifest severe cardiac fibrosis as a common hallmark [194,196]. More importantly, regulatory mechanisms controlling the translation initiation step are unexplored and need to be uncovered to provide additional therapeutic targets to silence ECM protein production and antagonize cardiac fibrosis.

#### 4.2.4. Editing-Defective Aars1 Mouse Shows Spontaneous Cardiac Proteinopathy and Fibrosis

In the previous sections, we discussed how quantitative changes in the global proteome or specific fibrotic proteins can affect cardiac function and contribute to heart disease. In addition to protein abundance, protein sequence and folding accuracy are also essential. Mistranslated or misfolded proteins are an emerging hallmark of multiple human diseases, including cardiac disease [197,198]. The accumulation of misfolded proteins leads to protein aggregation, resulting in cell dysfunction and death. In most cases, misfolded proteins originate from genetic mutations, where missense mutations in the exon of a specific gene lead to the misfolded product of that protein. However, misfolded proteins can also be generated by inaccurate translation [6,199,200].

In eukaryotic cells, translation is strictly supervised to ensure fidelity. The correct translation of proteins requires the addition of the correct amino acid to their tRNA by a specific aminoacyl-tRNA synthetase, and the selection of the correct aminoacyl-tRNA according to the elongation cycle. Adding the proper amino acid to the tRNA largely depends on the editing activity of aminoacyl-tRNA synthetases (ARSs), which ensures accurate cognate tRNA aminoacylation [199,201]. Although errors in aminoacylation are relatively low, even small decreases in the editing activity of ARS dramatically affect cell survival [202,203,204,205].

Deficiency in the editing activity of ARS has been found to produce misfolded proteins associated with several diseases, including heart disease. One example of this is the editing-defective alanyl-tRNA synthetase (AARS1), which is caused by the introduction of missense mutations in the editing domain of AARS1 [200] (Figure 7C). Cys^723^, an essential amino acid in the editing domain of AARS1, was mutated into Ala, leading to excessive production of mischarged Ser-tRNA^Ala^ instead of Ala-tRNA^Ala^, as demonstrated in vitro. When introduced in vivo, this editing-defective mutation resulted in the production of misfolded protein and increased cell death. Furthermore, *Aars1*^C732A/C732A^ mice were found to be embryonic lethal, while *Aars1*^C732A/+^ mice showed extensive age-related cardiac fibrosis and CM death. Although the mechanism behind these phenotypes is not fully understood, and no human editing function mutations have been identified, these animal studies suggest that editing activity is essential for the survival of multicellular organisms and provide evidence for the correlation between translation fidelity and cardiac disease in rodents.

## 5. Translation-Manipulating Therapeutics for Heart Disease Treatment

### 5.1. Translation-Targeted Medicines for Cardiac Disorders

Since elevated global mRNA translation was observed in cardiac remodeling [106,108,113], scientists have taken multiple measures to block cardiac protein synthesis for heart failure treatment. Contrary to the expectation, rabbits treated with puromycin, a global translation inhibitor, or fed with a protein-free diet manifested accelerated heart failure progression in a heart disease model [206]. These detrimental effects likely result from the robust translational inhibitory effect of puromycin and complete amino acid starvation, underscoring the need for mild and selective translation reduction strategies. Consistent with this concept, rapamycin, a small molecule inhibitor of mTORC1, can attenuate transverse aortic constriction-induced cardiac hypertrophy in mouse models by suppressing S6K [167], which implies that mild translational inhibition is a viable therapeutic strategy to counteract pathogenic cardiac remodeling. However, inhibition of the mTORC1 pathway can cause adverse immune-suppressant effects and may not be a safe approach for long-term anti-fibrotic treatment. Nevertheless, rapamycin has been tested in a phase I clinical trial to treat patients with heart failure with preserved ejection fraction (ClinicalTrials.gov ID: NCT04996719).

Following the idea of potential cardioprotective roles of mild translational inhibition in heart failure, additional translation inhibitors have been extensively tested in pre-clinical animal models. A prolyl-tRNA synthetase-specific inhibitor, halofuginone [207,208], is derived from an anti-malarial Chinese herbal medicine, Chang Shan. Halofuginone blocks the binding of proline and tRNA^Pro^ to (E)PRS and prevents the ligation of the amino acid to its cognate tRNA [207,208]. Halofuginone has been shown to reduce collagen expression [207] and skeletal muscle fibrosis in Duchenne Muscular Dystrophy (DMD) (HT-100, a delayed-release formulation of halofuginone from Akashi Therapeutics) in phase II clinical trials. Halofuginone protects the heart from multiple cardiac stresses in pre-clinical mouse heart failure models, including angiotensin II infusion, transverse aortic constriction-induced pressure overload, and ischemia-reperfusion [209]. Halofuginone has two significant cardioprotective effects: (1) it promotes cardiomyocyte autophagy and cell survival, and (2) it reduces extracellular matrix protein expression and inhibits cardiac fibrosis [209]. The therapeutic mechanism of halofuginone has been studied at the transcriptional level to trigger an amino acid starvation response by inhibiting the incorporation of proline into the protein synthetic chain [207,209,210]. Our group studied its direct impact on the translation of pro-fibrotic genes and anti-fibrotic effects in vivo [31]. General translation factors are well-established antibiotic targets for eradicating infectious microorganisms [178]. We proposed that mild translational inhibition using an EPRS1-specific inhibitor and genetic knockout of one allele of the *Eprs1* gene can effectively reduce cardiac remodeling and fibrosis by specifically inhibiting proline-rich mRNA translation, while preserving global mRNA translation (non-proline-rich genes). This new therapeutic approach, targeting evolutionarily conserved, ubiquitous translation factors, can be used to treat cardiac fibrosis of multiple etiologies and is generalizable to treating fibrosis in various organs.

In addition, the integrated stress response inhibitor (ISRIB), an eIF2B complex activator that blocks ISR, can protect hearts from ischemic stress and post-infarct atrial fibrillation [211]. In another study, ISRIB suppressed hyperlipidemia-induced inflammasome activation and inflammation, thereby reducing atherosclerosis [212]. Intriguingly, the mechanism of action for ISRIB is opposite to that of halofuginone in terms of activating the ISR. Rigorous clinical studies are necessary to evaluate the potential beneficial effects of ISRIB in patients with heart disease and to determine whether the cardioprotective benefit exists and results from the inhibition of ISR.

### 5.2. RNA Secondary Structure as a Potential Therapeutic Target for ASO Treatment of Cardiac Hypertrophy

**Figure 8:**
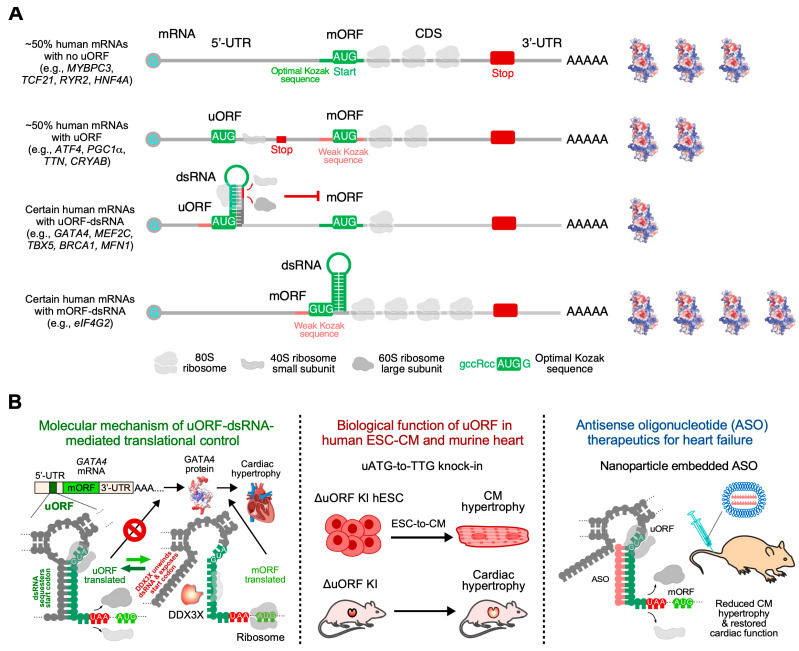
uORF-dsRNA modulates mORF translation of *GATA4* mRNA and cardiomyocyte hypertrophy. (**A**). About half of human mRNAs have no upstream open reading frames (uORFs) in the 5′-untranslated region (UTR), maximizing translation of housekeeping proteins such as sarcomere factors in cardiomyocytes (**first row**). About the other half of human mRNAs have at least one or more than one uORF that inhibits the main (m)ORF translation to limit the expression of dose-sensitive proteins (**second row**). Double-stranded (ds)RNA downstream of uAUG or mGUG synergizes with the AUG canonical or GUG noncanonical start codons to activate GATA4 uORF translation (**third row**) and mORF translation of eIF4G2 (**fourth row**). (**B**). **Left panel**: cis- and trans-acting molecular mechanisms regulating the translational balance between *GATA4* uORF and mORF. **Middle panel**: physiological function of uORF in human embryonic stem cell-derived cardiomyocytes (hESC-CM) and mice (using CRISPR-Cas9-mediated gene editing to introduce ATG-to-TTG knock-in mutation). **Right panel**: therapeutic intervention of cardiac hypertrophy by nanoparticle embedding antisense oligonucleotides (ASOs) targeting GATA4 uORF-dsRNA. uORF translation is enhanced while mORF translation is inhibited, thereby GATA4 protein level decreases, and isoproterenol- and transverse aortic constriction surgery-induced cardiac hypertrophy is compromised.

**Figure 9:**
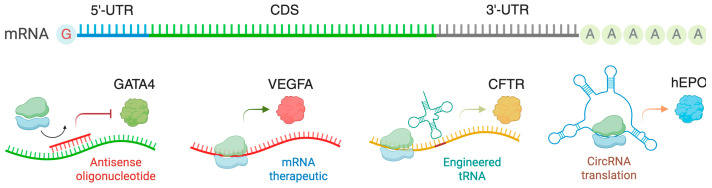
Potential RNA-based translation-manipulating therapeutic modalities for treating heart disease. Antisense oligonucleotides (ASO) can inhibit pathogenic protein translation and activate protective protein synthesis, such as GATA4, a pro-hypertrophic transcription factor. mRNAs can be delivered by a lipid nanoparticle to internal organs for replacement or regenerative therapy, such as *VEGFA* mRNA trial for treating MI patients and ongoing cancer vaccine clinical trials. Engineered tRNAs have the potential to treat genetic diseases caused by nonsense codons, including cystic fibrosis and liver metabolic disorders. circRNAs have the promise to complement mRNAs as a protein translation platform for RNA therapeutics development to prevent infectious disease and anemia.

The underlying molecular mechanisms governing uORF regulation within cells remain enigmatic. Our lab has identified a double-stranded RNA (dsRNA) structure within the *GATA4* 5′ uORF, enhancing uORF translation while concurrently inhibiting mORF translation [11] (Figure 8A,B). We demonstrated that antisense oligonucleotides (ASOs) designed to disrupt this dsRNA structure effectively promoted mORF translation. Conversely, ASOs designed to base-pair immediately downstream, forming a bimolecular double-stranded region of either the uORF or mORF start codon, enhanced the translation of their respective sequences (Figure 8B). In human embryonic stem cell (hESC)-derived CMs, employing GATA4 uORF-enhancing ASOs reduced levels of cardiac GATA4 protein and increased resistance to CM hypertrophy (Figure 8B and Figure 9), in line with the observed GATA4 loss-of-function phenotypes. Our findings extend beyond GATA4, demonstrating the broad applicability of uORF-dsRNA- or mORF-targeting ASOs in regulating translation for other mRNAs. This research unveils a novel regulatory paradigm that governs translational regulation, offering a valuable strategy for modulating protein expression and cellular phenotypes by selectively disrupting or forming dsRNA structures downstream of uORF or mORF start codons.

In the future, several remaining questions need to be addressed: (1) What distance and length rules govern the structural and functional features of uORF-dsRNA elements? (2) What trans-acting factors and pathological stress stimuli regulate uORF-dsRNA activity? (3) What is the in vivo significance of uORF and dsRNA elements in translating *GATA4* mRNA and cardiac pathophysiology? Answers to these questions will help us understand how uORFs are involved in downstream mORF translational control and how translational regulation of GATA4 (or other proteins such as MEF2C, TBX5, NKX2-5, etc.) is vital to normal development or pathogenic processes. Our recent findings reveal that a CRISPR-Cas9-mediated ATG-to-TTG mutation, followed by a PAM (protospacer adjacent motif) mutation (CTG-to-CTC), in a global knock-in mouse model, shows spontaneous cardiac hypertrophy without significant fibrosis after 6–7 months of age. Single-nucleus RNA-seq and ATAC-seq (Assay for Transposase-Accessible Chromatin) dual-omics analysis confirms the upregulation of GATA4 target gene transcription, including *Ttn*, *Tmx1*, *Myh6*, *Actc1*, and *Mybpc3* [213]. Interestingly, *GATA4* mRNA does not undergo nonsense-mediated mRNA decay (NMD) [214,215,216,217], as evidenced by the fact that uORF-inactivating knock-in mutation (uATG-to-TTG) in hESC-CM and the mouse genome does not increase *GATA4* mRNA expression. Nearly 50% of human mRNAs contain uORF, while NMD is observed to influence the expression of ~10% of human mRNAs, indicating a limited impact of NMD on uORF-containing mRNAs at the physiological level across the transcriptome at the baseline [218,219,220,221], compared to other mRNA decay pathways, such as miRNA-induced silencing complex [222,223,224] and codon optimality-mediated mRNA degradation [225,226,227].

Interestingly, a contemporary study has shown a similar uORF-dsRNA-mediated translation regulatory mechanism acting in plants and human cancer cells to control the protein synthesis of stress-responsive factors and tumor suppressor genes, respectively [228]. As an evolutionarily conserved molecular mechanism, similar observations have also been reported for alternative upstream translation initiation at near-cognate start codons in yeast [229] and repeat-associated non-AUG (RAN) translation of the GC-rich dsRNA formed by genetic expansion of gain-of-function toxic hexanucleotide GGGGCC repeats in human diseased brains [230].

### 5.3. Chemically Modified mRNA-Based CAR-T-Mediated Therapeutics for Cardiac Fibrosis

Cardiac fibrosis, indicated by stiffening and scarring of heart tissues, is a hallmark of most heart diseases and is considered a primary cause of mortality. Cardiac fibroblasts undergo proliferation in response to heart injury, such as acute myocardial infarction and chronic hypertension, and are subsequently activated to differentiate into myofibroblasts. Myofibroblasts secrete and deposit excessive amounts of extracellular matrix proteins, leading to increased stiffness and reduced compliance of the cardiac tissue. Prolonged cardiac fibrosis is a key contributory factor in the progression of various forms of cardiac disease and consequent heart failure. Currently, there are limited effective clinical interventions and therapies available to target fibrosis. A recent conceptual advance demonstrates the efficiency of reprogrammed T-cell immunotherapy in targeting pathological cardiac fibrosis in mice specifically [231]. Engineered mouse cardiac fibroblasts expressing a xenogeneic antigen protein can be effectively targeted and eradicated by transferring antigen-specific CD8-positive T cells. An endogenous target of cardiac fibroblasts, fibroblast activation protein (FAP), was discovered by transcriptome profiling-based gene signature analysis of heart tissues obtained from healthy controls, dilated cardiomyopathy, and hypertrophic cardiomyopathy patients. Adoptive transfer of T cells expressing a chimeric antigen receptor (CAR-T) against FAP results in a substantial reduction in cardiac fibrosis and restoration of left ventricular contractile function in mice treated with angiotensin II and epinephrine. These findings provide an innovative approach to developing immune therapies for treating heart disease.

The researchers can generate therapeutic CAR-T cells in vivo by introducing CD5-targeted lipid nanoparticles that bear pre-designed instructive mRNAs, which are required to reprogram T lymphocytes. Preclinical testing in a mouse model of heart disease demonstrated that the approach successfully reduced cardiac fibrosis and restored heart function. The potential to generate CAR-T cells in vivo using modified synthetic mRNAs may have many therapeutic applications. However, one of the caveats lies in the long-term side effects of CAR-T attacking physiological fibrosis processes such as wound healing and skeletal regeneration. To avoid potential systematic off-target effects and related toxicity, the same group of researchers developed an improved immunotherapy strategy to produce transient anti-fibrotic CAR-T cells that can recognize cardiac fibroblasts in mouse hearts by delivering chemically modified mRNA in T-cell-targeted lipid nanoparticles [232] (Figure 9). The efficacy of the reprogrammed CAR-T cells was evaluated in vivo by injecting CD5-targeted lipid nanoparticles into a mouse model of heart failure induced by a neurohumoral agonist. Efficient lipid nanoparticle-directed delivery of modified mRNA encoding the CAR to T lymphocytes generated transient but effective CAR T cells in animals. These CAR-T cells promoted trogocytosis and retained the specific target antigen protein as they accumulated in the spleen. Treatment with engineered mRNA-targeted lipid nanoparticles reduced fibrosis and partially improved cardiac function in mouse models of heart failure. As a generalizable therapeutic platform, this method of in vivo generation of CAR-T cells is promising for treating various human diseases related to organ fibrosis. At the technical level, more strategies need to be developed to further increase translation efficiency and allow minimal mRNA to produce the maximum amount of therapeutic proteins, such as multi-capped mRNA technology [233]. Although the CAR-T therapy has been approved and extensively applied in treating leukemia, future clinical trials need to be rigorously conducted for repurposing it for treating cardiac fibrosis and heart disease, with serious consideration of potential off-target side effects that negatively impact connective tissue regeneration.

### 5.4. Chemically Modified mRNA-Based Therapeutics for Ischemic Heart Disease

mRNA carries genetic codon-based instructions transcribed by RNA polymerases from DNA, which are then translated by ribosomes to produce proteins. Proteins are the primary bioactive molecules directing various biochemical and biological functions in cells. Pfizer and Moderna’s pioneering mRNA therapeutics are designed to utilize cellular translation machinery to produce specific medicinal proteins (Figure 9), such as the VEGF-A protein. Vascular endothelial growth factor A (VEGF-A) is a paracrine cytokine required for new blood vessel formation. It can stimulate the proliferation of epicardial progenitor cells, which differentiate into endothelial cells that facilitate heart repair by promoting angiogenesis and blood flow. Therefore, VEGF-A has therapeutic effects on the cardiovascular system; however, insufficient delivery is a significant challenge that impedes its clinical development. AstraZeneca and Moderna have developed *VEGF-A* mRNA therapeutics for treating patients with myocardial infarction. A Phase II clinical study, EPICCURE, was completed using modified mRNA encoding VEGF-A (AZD8601) without lipid encapsulation for injection into the hearts of patients with reduced left ventricular ejection fraction and coronary artery bypass surgery [234]. AZD8601 met the primary endpoint of safety and tolerability in heart failure patients with no arrhythmias, mortality, and mRNA-triggered immune responses. Positive outcomes suggest improvement in left-ventricular ejection fraction and pro-B-type natriuretic peptide levels. However, this study is limited in size, and future clinical trials are required to confirm efficacy and safety with a larger patient cohort.

Cardiomyocyte-specific overexpression of CCND2 (cyclin D2) has been reported to promote recovery from cardiac injury in mammalian models of heart failure triggered by myocardial infarction. Modified mRNA of *CCND2* was recently reported to activate the cell cycle of CMs in hearts following myocardial infarction injury in small and large mammalian hearts, such as mice and pigs [69]. The CM-specific modified *CCND2* mRNA translation system (CM SMRTs) contains two modRNA modules. One modRNA encodes CCND2 protein and bears an L7Ae-binding site. The other modRNA encodes L7Ae (an archaeal large ribosomal protein that recognizes and binds to kink-turn motifs in RNA) and possesses the specific recognition elements for the CM-specific microRNAs miR-208 and miR-1. Therefore, L7Ae is silenced by highly abundant endogenous miR-1 and miR-208 in CMs, while it inhibits *CCND2* mRNA translation in non-CM cells. This bidirectional regulatory effect from the modRNA drives CM-specific CCND2 expression. Intramyocardial injections of the CM *CCND2* SMRTs significantly enhanced CM proliferation and reduced infarction in both MI models of mice and pigs. They increased the left ventricular ejection fraction in animals compared to the control group with myocardial infarction surgery. The long-term goal is to advance mRNA technology and the ability to engineer different mRNAs as payloads to produce therapeutic proteins for disease treatment.

## 6. Concluding Remarks and Future Perspective

**Figure 10:**
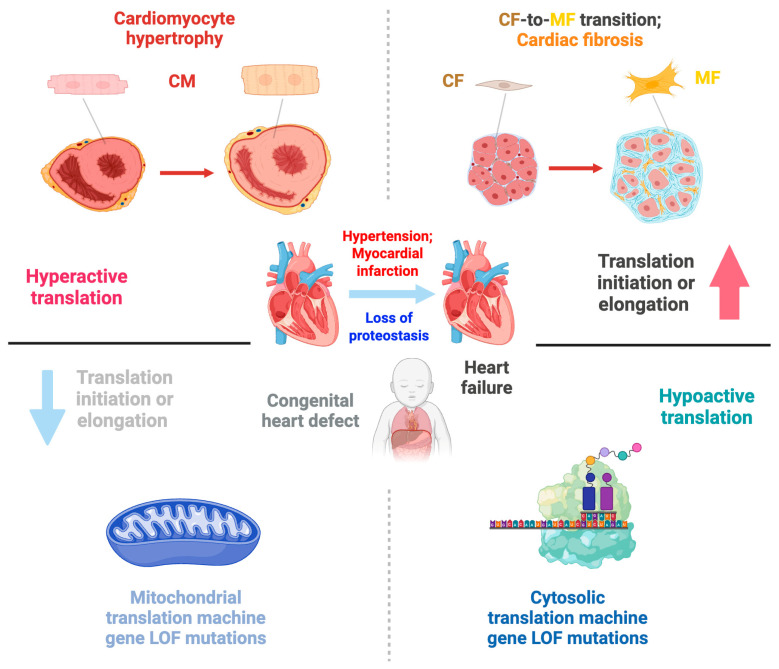
Overall schematic of the etiology of translation dysregulation in heart disease. **Top panel**: adult heart failure caused by cardiac pathological remodeling, including cardiac hypertrophy and fibrosis, triggered by enhanced translation initiation and elongation at global and transcript-selective levels in both cardiomyocytes and cardiac fibroblasts. **Bottom panel**: congenital heart diseases or early onset cardiomyopathy driven by loss-of-function (LOF) genetic mutations in cytosolic or mitochondrial translation machine component genes (e.g., aminoacyl-tRNA synthetases, ribosome proteins, translation initiation or elongation factors, RNA helicases, among other RNA-binding proteins). These developmental defects in the cardiovascular system result from reduced translation at global and transcript-selective levels during cardiac cell proliferation, differentiation, or migration.

Along with transcription and epigenetic regulation, signal transduction, the sarcomere network, and protein modifications and degradation, translational control plays a crucial role in maintaining cardiac health and preventing disease. When the translation program is dysregulated, a variety of heart diseases will manifest or progress (Figure 10). Hyperactive translation at the global transcriptome level, or in a specific cohort of mRNAs encoding pathogenic proteins in cardiomyocytes and cardiac fibroblasts, promotes cardiac hypertrophy and fibrosis, which drive the progression of pathological cardiac remodeling and heart failure (Figure 10). Conversely, hypoactive translation activity in the fetal heart, often caused by loss-of-function genetic mutations in translation machinery components, leads to decreased cardiac cell proliferation and differentiation, causing developmental defects during embryonic stages and resulting in congenital heart disease. Increased or reduced translation in heart failure and congenital heart disease can happen at the initiation or elongation step (Figure 10). Therefore, understanding how translational control operates in pathological cardiac remodeling has significant implications for developing new treatments to combat heart diseases.

Global translation inhibition, such as using mTOR inhibitors, can reduce cardiomyocyte hypertrophy and fibrosis by silencing the production of pro-hypertrophic and pro-fibrotic proteins involved in sarcomere and extracellular matrix formation. However, potent inhibition of protein synthesis may lead to unpredictable cytotoxicity and side effects. To achieve a safer therapeutic approach, targeting specific translation factors or RBPs is essential to suppress the translation of pathogenic factors, as well as to optimize dosage and treatment duration. Alternatively, transcript-specific translational regulation can be targeted for manipulation of protein synthesis for one or multiple selected mRNAs, further reducing unintended off-target effects. For instance, ASO can be designed to bind precisely to a unique site on target mRNAs, thereby preventing specific RBPs from interacting with them. This approach modulates the translation efficiency of the targeted mRNA directly (blocking translation initiation of oncogenic mRNAs) or indirectly (regaining an exon to prevent premature translation termination) for potential therapeutic purposes [15,235]. Although translation-manipulating ASO is a promising strategy, this approach is still in the early stages of research and development. Future studies are required to improve ASO design, ensure target mRNA specificity, and overcome challenges in cell-type-specific delivery, as well as long-term safety and efficacy. 

Among more than a thousand classical RBPs, specific cohorts can bind to either single-stranded RNA (e.g., linear motifs) or double-stranded RNA (e.g., structural elements). Secondary and tertiary structures formed by dsRNA elements are critical in RNA processing, stability, translation, and interaction with RBPs. Understanding the structure of RNA can provide valuable insights into RNA-protein interactions and the mechanisms of RBP-mediated translational regulation. It also guides the design, screening, and development of RNA-based therapeutic compounds, including ASOs, siRNAs, miRNAs, and synthetic mRNAs. RNA structures can be determined through the chemical probing methods such as SHAPE-MaP (Selective 2′-Hydroxyl Acylation analyzed by Primer Extension and Mutational Profiling), which measures RNA flexibility and conformation at a single-nucleotide resolution. For example, in single-stranded regions, 2-methylnicotinic acid imidazolide (NAI) forms an adduct with free 2′-OH groups on the RNA sugar backbone. Adduct formation occurs at a rate of 1–3% and further induces cDNA mutations during reverse transcription. Mutation rates above DMSO control are calculated into reactivity scores that guide RNA folding using publicly available web tools, such as RNAStructure or RNAFold. Thus, a comprehensive atlas of RNA secondary structures can be drawn as a blueprint for identifying target sites for drug targeting to manipulate mRNA translation. For instance, precise transcriptome-wide mapping of uORF-dsRNA elements is needed in human or mouse cells to uncover novel therapeutic targets for ASOs or siRNAs. Based on Ionis’ pioneering work and our studies [11,236], novel translation-manipulating ASO-based therapeutics can be developed for treating diseases across multiple organs, including the heart and blood vessels. However, a significant hurdle for RNA-based treatment of cardiovascular disease is the cardiac cell type-specific delivery of RNA therapeutics. There is an urgent need to develop novel RNA drug delivery tools with high uptake efficiency, cell-type specificity, low immunogenicity, and minimal interference with RNA drug efficacy, such as lipid nanoparticles and RNA aptamers [237,238,239]. In the long term, we aim to generalize the application of translation-manipulating ASOs across various cell types, organs, and disease models and conjugate ASOs to carrier RNA aptamers to achieve cell-type-specific delivery.

In addition to ASO [11,236], multiple promising RNA-based translation-modulating therapeutic strategies are emerging, including tRNA [240], circRNA (circular RNA) [241], lncRNA (long noncoding RNA) [242], SINE-up element [243], among others. Anticodon-engineered tRNA enables ribosome recoding of premature termination codons to treat genetic diseases, such as cystic fibrosis caused by nonsense mutations in the cystic fibrosis transmembrane conductance regulator gene (*CFTR*) [240] (Figure 9). Learning from the lesson of failure in a pre-clinical testing of tRNA therapy for targeting hemophilia A and shutdown of the HC Bioscience, further mechanistic studies and advanced delivery tools are needed to confirm the safety of tRNA-mediated readthrough of premature termination codons across the transcriptome, while avoiding the decoding of native stop codons and off-target side effects on other cell types, to prevent the production of potentially harmful C-terminal extended proteins in both target and non-target cell types. As an emerging novel RNA therapeutic modality, circRNA is currently in preclinical testing as a more effective protein expression platform than linear mRNAs, due to its high stability, long half-life in serum and cells (lacking 5′-cap or 3′-polyA tail, which are targeted by exoribonucleases), and its potential for endless translation—possibly bypassing ribosome recycling once loaded through an engineered internal ribosome entry site (start and stop codons are close together for continuous ribosome reinitiation after translation termination without drop-off) [241]. In a recent proof-of-principle preclinical trial, circRNA has been used to express a secreted protein, human erythropoietin (hEPO), to treat anemia in a mouse model [241] (Figure 9). However, it remains unclear whether the internal ribosome entry site structure remains stable upon delivery into target cells and whether it can efficiently recruit ribosomes to initiate translation adequately to produce therapeutic proteins at a sufficient level compared to a linear mRNA in humans.

Intracellular protein homeostasis is crucial for maintaining optimal cellular function, and disruptions can lead to the development of pathological conditions. Maintaining protein homeostasis involves a balanced process of protein synthesis, post-translational processing, and protein degradation. Accumulating evidence indicates that coordinating translational control with protein degradation pathways—such as ribosome-associated quality control (RQC) [244], eIF2α phosphorylation-dependent autophagy [83], and mTORC1-dependent regulation of translation and autophagy [245]—is essential for cardiac protein homeostasis. Ribosome stalling caused by abnormal translation elongation results in the production of truncated proteins and activates RQC. Although research increasingly links RQC dysfunction to neurological disorders, cancer, developmental disorders, and metabolic diseases [246], its role in heart biology and disease remains largely unexplored. A loss-of-function mutation in the ribosomal protein RPL3L, which is specifically expressed in the heart and skeletal muscle, has been shown to slow the translation of specific codons for proline and alanine, resulting in ribosomes colliding along the mRNA. These collisions may lead to misfolded proteins related to cardiac muscle contraction, which are then degraded by RQC. Functionally, RPL3L deficiency results in impaired heart contractility and neonatal dilated cardiomyopathy [51]. As we advance, studying RQC and related E3-ubiquitin ligases, such as RNF10—responsible for ubiquitination of RPS3 and the degradation of stalled 40S ribosomal subunits—will be necessary for a better understanding of the etiology of heart diseases [247,248].

During ribosome stalling and collision, de novo synthesized polypeptide chains can misfold and aggregate, resulting in unfolded protein stress. Autophagy degrades misfolded proteins and aberrant protein aggregates to reduce proteotoxic stress and provide metabolic reserves. Phosphorylation of eIF2α by protein kinase R-like ER kinase (PERK) inhibits eIF2α-GDP recycling to eIF2α-GTP, thereby suppressing overall translation; however, this mechanism selectively promotes the translation of autophagy-associated activating transcription factor 4 (ATF4) via a uORF-dependent mechanism [249]. Thrombospondin 1 (THBS1), which activates ER stress, mediates PERK-eIF2α-ATF4-induced autophagy, leading to cardiac atrophy and lethal cardiomyopathy [250]. Melatonin reduces the interaction between vascular endothelial growth factor-B (VEGF-B) and glucose-regulated protein 78 (GRP78), as well as between GRP78 and PERK, which increases PERK phosphorylation and induces PERK-eIF2α-ATF4-mediated autophagy. As a result, melatonin alleviates diabetic cardiomyopathy, reduces cardiac hypertrophy and fibrosis [251], and decreases CM apoptosis while protecting the heart from MI injury by increasing eIF2α phosphorylation [252]. This highlights the critical role of eIF2α phosphorylation in managing misfolded and unfolded protein stress in the heart. Besides its function in protein synthesis, mTORC1 negatively regulates autophagy by modulating downstream autophagy-initiating kinase 1 (ULK1) and ATG13, orchestrating the delicate balance between anabolic and catabolic pathways necessary for maintaining cardiac protein homeostasis. AMP-activated protein kinase (AMPK) directly phosphorylates ULK1 to promote autophagy [253]. Activation of AMPK has been shown to enhance autophagy in hearts under pressure overload, resulting in delayed cardiac hypertrophy and improved cardiac function [254]. AMPK also activates autophagy and protects the heart against diabetes and ischemic injury [255,256]. Similar effects were observed with the use of metformin, a commonly prescribed anti-diabetic drug and AMPK activator [257,258].

As another future research direction, a deeper understanding of how translational control interfaces with protein degradation pathways may uncover new regulatory circuits vital for cardiac protein homeostasis and disease mechanisms, potentially aiding in the development of more effective and precise therapies for cardiac conditions like HFpEF [196,259]. Li, C et al. provide an exemplary mechanism of a noncanonical IRE1 (Inositol-requiring enzyme 1)-eIF4G1-eIF3 translation initiation complex involved in transcript-selective translational control of epidermal growth factor receptor through binding of a 5′-UTR terminal oligopyrimidine motif. This work suggests a novel connection among the unfolded protein response, protein degradation, and mRNA translation [260]. Investigating translational control mechanisms in the development and progression of metabolic syndrome, diabetic cardiomyopathy, and HFpEF is crucial for identifying novel therapeutic targets and developing innovative treatment strategies. Additionally, exploring the translation landscape across different cardiac and vascular cell types (e.g., endothelial cells, smooth muscle cells, and immune cells) and during crosstalk among various organs (such as the heart, lung, kidney, liver, and brain) will emerge as a prosperous area for multidisciplinary research and systematic investigation, facilitated by extensive collaborations within a diverse scientific community sharing common interests and complementary expertise.

## Data Availability

No new data were created or analyzed in this study.
